# Dichotomous intronic polyadenylation profiles reveal multifaceted gene functions in the pan-cancer transcriptome

**DOI:** 10.1038/s12276-024-01289-w

**Published:** 2024-10-01

**Authors:** Jiao Sun, Jin-Young Kim, Semo Jun, Meeyeon Park, Ebbing de Jong, Jae-Woong Chang, Sze Cheng, Deliang Fan, Yue Chen, Timothy J. Griffin, Jung-Hee Lee, Ho Jin You, Wei Zhang, Jeongsik Yong

**Affiliations:** 1 Department of Computer Science, University of Central Florida, Orlando, FL 32816 USA; 2 Department of Biostatistics, St. Jude Children’s Research Hospital, Memphis, TN 38105 USA; 3 Department of Pharmacology, Chosun University School of Medicine, Gwangju, 61452 Republic of Korea; 4 Department of Cellular and Molecular Medicine, Chosun University School of Medicine, Gwangju, 61452 Republic of Korea; 5 Department of Biochemistry, Molecular Biology and Biophysics, University of Minnesota Twin Cities, Minneapolis, MN 55455 USA; 6 Department of Electrical and Computer Engineering, Johns Hopkins University, Baltimore, MD USA; 7 Present Address: SUNY Upstate Medical University, Syracuse, NY 13210 USA

**Keywords:** Gene regulatory networks, Genetics research, Gene expression

## Abstract

Alternative cleavage and polyadenylation within introns (intronic APA) generate shorter mRNA isoforms; however, their physiological significance remains elusive. In this study, we developed a comprehensive workflow to analyze intronic APA profiles using the mammalian target of rapamycin (mTOR)-regulated transcriptome as a model system. Our investigation revealed two contrasting effects within the transcriptome in response to fluctuations in cellular mTOR activity: an increase in intronic APA for a subset of genes and a decrease for another subset of genes. The application of this workflow to RNA-seq data from The Cancer Genome Atlas demonstrated that this dichotomous intronic APA pattern is a consistent feature in transcriptomes across both normal tissues and various cancer types. Notably, our analyses of protein length changes resulting from intronic APA events revealed two distinct phenomena in proteome programming: a loss of functional domains due to significant changes in protein length or minimal alterations in C-terminal protein sequences within unstructured regions. Focusing on conserved intronic APA events across 10 different cancer types highlighted the prevalence of the latter cases in cancer transcriptomes, whereas the former cases were relatively enriched in normal tissue transcriptomes. These observations suggest potential, yet distinct, roles for intronic APA events during pathogenic processes and emphasize the abundance of protein isoforms with similar lengths in the cancer proteome. Furthermore, our investigation into the isoform-specific functions of JMJD6 intronic APA events supported the hypothesis that alterations in unstructured C-terminal protein regions lead to functional differences. Collectively, our findings underscore intronic APA events as a discrete molecular signature present in both normal tissues and cancer transcriptomes, highlighting the contribution of APA to the multifaceted functionality of the cancer proteome.

## Dichotomous intronic APA effects: unveiling proteome programming in cancer

Understanding our genes is vital for combating diseases like cancer. A crucial gene expression process is alternative polyadenylation. These versions can influence cell behavior and are associated with various diseases, including cancer. The role of a specific APA type, intronic APA, in cancer was unclear. This study examined intronic APA’s effect on cancer by analyzing cancer patient data. They found that intronic APA profiles vary greatly between normal and tumor tissues across different cancer types, indicating that intronic APA plays a complex role in cancer biology. The results showed that intronic APA contributes to the diversity of mRNA endings in cancer, affecting gene expression. This could lead to new diagnosis or treatment approaches. The researchers concluded that intronic APA is a key factor in cancer’s molecular landscape, providing new insights into cancer development and progression.

This summary was initially drafted using artificial intelligence, then revised and fact-checked by the author.

## Introduction

Pre-mRNA processing, involving capping, splicing, and polyadenylation, is crucial for mRNA maturation^1^. Polyadenylation, mediated by multisubunit machinery using the poly(A) signal (PAS), involves the addition of a poly(A) tail to mRNA. Over 70% of human genes with multiple PASs exhibit alternative cleavage and polyadenylation (APA)^2–4^. APA events occur in the 3’-untranslated region (3’-UTR) or intronic region, resulting in UTR-APA or intronic APA/coding region APA, respectively^5^. UTR-APA produces mRNA isoforms with varying 3’-UTR lengths, influencing mRNA stability, localization, and interactions with regulatory elements, such as RBPs and miRNAs^6^. In contrast, intronic APA events generate transcript isoforms that potentially yield C-terminal truncated proteins, significantly impacting cellular functions^7,8^.

APA is also tissue specific^9^ and is globally regulated across different cellular contexts through signaling pathways and environmental stresses or stimuli^2^. For instance, activation of mammalian target of rapamycin (mTOR) induces widespread shortening of mRNA 3’-UTRs via UTR-APA, enhancing protein synthesis from these shortened transcripts^10^. These UTR-APA transcripts also serve as a defense mechanism against ER (Endoplasmic Reticulum) stress^11^. However, it remains unclear whether physiological cues can mediate intronic APA.

Deregulation of APA has been implicated in various human diseases, including cancer, metabolic disorders, and neurological diseases^7,12,13^. Cancer cells exhibit global 3’-UTR shortening of transcripts^14,15^, along with increased transcripts featuring intronic APA, resulting in the activation of oncogenes and suppression of tumor suppressors^8^. Although the expression of cleavage/polyadenylation factors and other RNA-binding proteins (RBPs) has been implicated^16^, the precise mechanisms by which *trans*-acting factors influence APA dynamics in cancer remain unclear. Profiling analyses have identified transcriptome-wide intronic APA-mediated truncated transcripts in chronic lymphocytic leukemia cells^8^. In multiple myeloma cells, reduced intronic APA transcript expression in plasma cells is correlated with shorter progression-free survival^8,17^, emphasizing the need for further understanding of the role and mechanisms of APA in cancer. Intronic APA is also critical for immune cell development and diversification^17^. Truncated transcript isoforms resulting from intronic APA, such as the intronic APA of the immunoglobulin M (IgM) heavy chain, produce secreted truncated IgM heavy chains lacking the membrane receptor domain, suggesting that intronic APA may serve as a regulatory mechanism to diversify the immune proteome^17^.

In this study, we investigated the significance of intronic APA in cancer by employing a custom-designed bioinformatics workflow. Surprisingly, our findings revealed the presence of dichotomous intronic APA profiles in all examined cancer types, displaying enrichment either in normal tissues or tumor samples. This comprehensive atlas of intronic APA events sheds light on previously unrecognized roles of C-terminal dynamics in the cancer proteome, emphasizing the significance of considering isoform identity in cancer-related studies.

## Materials and methods

### Selected reaction monitoring (SRM) for targeted mass spectrometry

For peptide detection using LC-SRM, WT and TSC1^-/-^ MEFs were washed, resuspended in PBS and lysed by sonication. The protein content was estimated using the Bradford assay. The samples were digested using the FASP protocol^18^ on 10-kDa MWCO filters (Pall nanosep) using trypsin (Promega). The resulting peptides were desalted using Strata-X columns (Phenomenex). C-terminal sequences of interest were generated as described in the section on the preparation of the in silico protein database. These sequences were imported into Skyline software^19^, where an in silico trypsin digest produced the expected precursor and fragment m/z values. Peptides were assumed to have a + 2 charge unless they contained histidine, in which case a 3+ charge was assumed. These mass lists were imported into Analyst software (Sciex) and used to generate unscheduled SRM methods on a Qtrap 5500 (Sciex). The dwell time for each transition was 20 ms, allowing a maximum of 75 transitions per injection. Repeated injections were required to test all possible peptides. The QTRAP 5500 was equipped with an Agilent 1100 capillary LC system operating at 8 µL/min. Solvents A and B were 98% H_2_O, 2% ACN + 0.1% formic acid, and ACN + 0.1% formic acid. A three-step gradient was used to separate the peptides. The column was 100 × 0.3 mm, with 2.7 µm HALO C18 particles with a 90 Å pore size (Eksigent). The digested samples were reconstituted to 1 µg/µL, and 2 µL was injected per analysis. The SRM data were imported into Skyline for manual quality assessment.

### Preparation of the in silico protein database

For all the detected truncated transcripts annotated in mm10 RefSeq, the nucleotide sequences of the last coding exons were translated into amino acid sequences and used as the candidate protein sequences for the SRM analysis. For the unannotated truncated transcripts identified using the integration method, the nucleotide sequence in the intron accumulation region and the exon before that intron were translated into amino acid sequences in all three possible reading frames. The sequences with a stop codon in the exon region or that only contained 6 bp after the exon were deleted. The remaining proteins were considered candidate protein sequences for the SRM analysis. Next, all the candidate protein sequences were cut after lysine (K) and arginine (R) to simulate trypsin-digested peptide sequences. The sequences containing methionine (M) or less than 7 amino acids were deleted. Then, we sorted the sequences based on their charge state and considered only the short peptides with charge states greater than 1 in the analysis. The charge state was calculated as follows:

$${\rm{Charge}}\;{\rm{state}}=\,{\#}\,{\rm{of}}({\rm{K}})+\,{\#}\,{\rm{of}}({\rm{R}})+\,{\#}\,{\rm{of}}({\rm{H}})+1$$

### Datasets

The TCGA transcript expression data were downloaded from UCSC Xena^20^. We focused on only 10 cancer types with a considerable number of normal samples (~10% of tumor samples). The 10 tumor types were BRCA, COAD, HNSC, KIRC, LIHC, LUAD, LUSC, PRAD, STAD, and THCA. The RNA-seq BAM files of BRCA samples were downloaded from dbGaP. We first ran TopHat2 to align the RNA-seq fastq files to the genome (hg38 annotation) and then used SAMtools^21^ to generate the read coverage profile. Clinical data for 10 cancer types were downloaded from cBioPortal. RNA-seq datasets generated using the cell lines are available in the BioProject repository under the following accession numbers: PRJNA886626, PRJNA944374 and PRJNA279582.

### Determination of intronic APA events

To identify and quantify intronic APA events, protein-coding transcripts of a gene are categorized as intronic APA transcripts and full-length transcripts using annotation. A transcript is categorized as an intronic APA if it satisfies the following two conditions: (1) its coding end is not the maximum coding end in the gene, and (2) its transcript end is not the maximum transcript end in the gene. We considered a total of 12,453 genes in the human genome for intronic APA events in the GENCODE annotation. There were 46,481 intronic APA transcripts and 46,996 full-length transcripts from these 12,453 genes. Genes with TPM < 1 in all the samples were filtered out from the data analysis. The transcript isoform expression in TPM was used to calculate the truncation ratio (TR) of a gene. The TR of intronic APA events was calculated using the following equation:

TR = [quantity of intronic APA transcript]/[quantity of total transcript (intronic APA + full-length)], where the quantity of intronic APA transcripts is the summation of all intronic APA transcript expressions from a gene. To identify conserved intronic APA events enriched in tumors and normal tissue of each type of cancer, we first applied a t test to assess the TR values of each gene in each cancer type. Then, the mean TR of all tumor samples or normal tissues in each cancer type was calculated. A higher TR mean in tumor samples with a t test *p* < 0.01 indicates a significant intronic APA event in the tumor samples. Significant intronic APA events in normal tissue samples were identified in a similar manner. In addition, the mean TR of normal tissues was used as a reference to determine the common significant intronic APA events in each cancer type based on the list identified above. If at least 80% of tumor samples showed a greater TR of a given gene compared to the mean TR in normal tissues, we considered such an intronic APA event from the gene to be a significant and common APA event in tumors. If at least 80% of tumor samples showed a lower TR of a given gene compared to the mean TR in normal tissues, we considered such an intronic APA event from the gene to be a significant and common APA event in normal tissues.

### Enrichment analysis

KEGG pathway enrichment analysis of genes with cancer-specific intronic APA events was performed using DAVID Bioinformatics Resources 6.8^22^.

### Identification of Pfam domains affected by intronic APA events

The protein sequences that were missing in the truncated proteins compared to the full-length protein were collected and defined as differential protein sequences. Potential Pfam domains existing in these differential protein sequences were surveyed using PfamScan^23^.

### Survival analysis using the log-rank test and Cox model

A Cox proportional hazards model with elastic net implemented with scikit survival was used for survival prediction. The Python package lifelines (ref: https://zenodo.org/record/3787142#.XrlRZZNKi9Y) was used to generate the KM plots. The genes with low variance (<1) across samples were removed before feature selection. The data were split into training (80%) and test (20%) sets. Then, a log-rank test was applied to select significant genes with a *p* value less than 0.05 on the training set of gene expression data and the TR of genes, separately. The integrated dataset concatenated gene expression and TR together. The high-risk and low-risk groups were determined using the prognostic index (PI) on the test set. The PI is the linear component of the Cox model and calculated as follows: ${\rm{PI}}={\sum }_{i=1}^{n}\beta {x}_{i}$, where *x*_*i*_ is the value of covariate i (n covariates in total), and its risk coefficient β was estimated from the Cox model fitted on the training set. The high-risk and low-risk groups were generated for the KM plot by splitting the ordered PI with an equal number of samples in each group.

### Clinical outcome prediction

We used SVM to implement clinical outcome predictions in TCGA BRCA patients for hormone receptor phenotypes (ER, HER2, PR, and triple negative) using the Python package scikit-learn (ref: https://scikit-learn.org/stable/about.html#citing-scikit-learn). Genes with low variance (<1) in the gene expression data were removed, and genes with low variance (<0.01) in the TR were removed. The top 100 significant features determined by the t test *p* value were selected from the training sets of gene expression and TR, separately. The test set (20%) is independent of the training data.

### Analyses of transcript and protein isoform lengths

Relative length changes in transcript isoforms due to intronic APA events were calculated as a fraction of 100%. The transcript with the most distal coding end and maximum coding length was defined as the full-length isoform in each gene. The position of the 3’-most exon in the intronic APA transcript was used to calculate the relative length of the intronic APA transcript. Only coding regions were counted in the calculation. Changes in protein length due to intronic APA events were calculated based on the number of amino acids encoded by each annotated APA isoform, including the full-length transcript isoform.

### Soft agar colony formation assay

Soft agar assays were performed in 6-well plates. The base layer of each well consisted of 2 ml with final concentrations of 1× medium and 0.6% low melting point agarose (Duchefa, Haarlem, The Netherlands). The plates were chilled at 4 °C until the solution solidified. Then, a 1-ml growth agar layer consisting of 4 × 10^4^ MCF-7 cells suspended in 1× medium and 0.3% low melting point agarose was added. The plates were again chilled at 4 °C until the growth layer congealed. An additional 1 ml of 1x medium without agarose was added on top of the growth layer. The cells were allowed to grow at 37 °C and 5% CO_2_ for 14 days, and the total colonies were stained with 0.005% crystal violet (Sigma‒Aldrich) and counted. Images were analyzed using Image-Pro Plus 4.5 software (Media Cybernetics, Silver Spring, MD). The assays were repeated three times.

### Cell migration assays

In vitro cell migration assays were performed in a 24-well Transwell plate with 8-mm polyethylene terephthalate membrane filters (BD Biosciences, Bedford, MA) separating the lower and upper culture chambers. The cells were grown to subconfluency (~75−80%) and serum-starved for 24 h. After detachment with trypsin, the cells were washed with PBS and resuspended in serum-free medium; then, the cell suspension (2 × 10^4^ cells) was added to the upper chamber. Complete medium was added to the bottom wells of the chamber. The cells that had not migrated were removed from the upper face of the filters using cotton swabs, and the cells that had migrated to the lower face of the filters were fixed with 4% formaldehyde and stained with 0.1% crystal violet. Images of three random 10x fields were captured from each membrane, and the number of migratory cells was counted. The mean of triplicate assays for each experimental condition was used.

### JMJD6LF and JMJD6S constructs

Human GFP-tagged JMJD6L (NM_001081461) and JMJD6S (NM_015167) ORF cDNA clones were purchased from OriGene Technologies (OriGene, USA).

## Results

### Alterations in cellular mTOR activity contribute to the dichotomous pattern of intronic APA

**Fig. 1:**
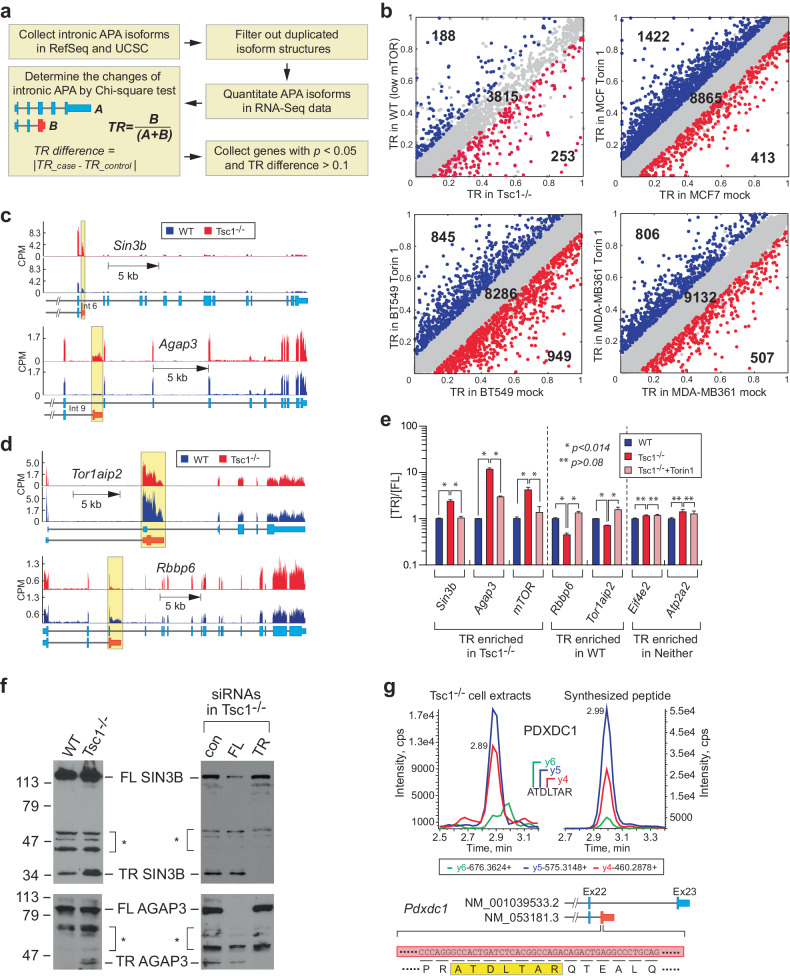
Bidirectional profile of intronic APA (alternative polyadenylation) events in response to changes in mTOR signaling in cells. **a** A schematic representation of the profiling of intronic APA events using RNA-seq data from cell lines. The RefSeq and UCSC gene structures were merged, and the RNA-seq data were quantified based on the collected structures. The chi-square test was used to determine significant intronic APA events between the control and the case based on the truncation ratio (TR), which was calculated as follows: TR = [quantity of intronic APA transcript] / [quantity of total transcript (intronic APA + full-length)]. The intronic APA events with a chi-square test *p* < 0.05 and TR difference >0.1 were considered significant events. **b** Scatter plots of the TRs of genes with low and high mTOR expression in cells. The analyses included wild-type (WT; low mTOR) and *Tsc1*^*-/-*^ (high mTOR) MEFs as well as the breast cancer cell lines MCF7, BT549, and MDA-MB-361. Cells were treated with 100 nM Torin 1 for 24 h to inactivate mTOR signaling in these cells. **c**, **d** Examples of RNA-seq read alignments of genes showing enriched intronic APA events in high-mTOR-*Tsc1*^*-/-*^ MEFs and low-mTOR-WT MEFs, respectively. The alignments are color-coded to highlight the regions with intronic APA events. **e** Real-time quantitative PCR (RT‒qPCR) validation of genes showing dynamic intronic APA events upon changes in mTOR signaling in cells. The analyses included WT, *Tsc1*^*-/-*^, and *Tsc1*^*-/-*^ cells treated with Torin 1 (100 nM, 24 h). The Y-axis scale is presented on a log scale. Four technical repeats were conducted, and Student’s *t* tests were performed for statistical analysis. The data are presented as the mean (SD). **f** Validation of protein expression of intronic APA mRNAs in WT and *Tsc1*^*-/-*^ MEFs. (Left panel) Full-length (FL) and truncated (TR) isoforms of SIN3B and AGAP3 were detected using western blotting. (Right panel) Confirmation of FL and TR isoforms using RNAi. RNAi-mediated knockdown of SIN3B and AGAP3 FL or TR isoforms in *Tsc1*^*-/-*^ MEFs. **g** A mass spectrometry approach, namely, selected reaction monitoring (SRM), was used to detect TR isoforms in the *Tsc1*^*-/-*^ proteome. A peptide for TR PDXDC1 is highlighted as an example, with the peptide sequence originating from exonized intron sequences in the intronic APA transcript highlighted in the yellow box. The gene structure of the intronic APA transcript of *Pdxdc1* is also shown. Fragmented ion spectra for the C-terminus of PDXDC1 from *Tsc1*^*-/-*^ cell extracts and synthesized peptide are shown as an example.

In our previous study, we discovered that the activation of mTOR is linked to the widespread occurrence of 3’-UTR APA events, resulting in increased expression of transcript isoforms with shortened 3’-UTRs throughout the transcriptome^10^. To explore another type of APA occurring within intron regions, namely, intronic APA events, we devised a workflow that leverages existing RNA-seq datasets and genome annotations, specifically RefSeq^24^ and UCSC^25^. This workflow enables the quantification of intronic APA and full-length transcript isoforms, allowing us to compute the truncation ratio (TR) by comparing the expression of the APA isoform to the total transcript amount (Fig. 1a).

In our analysis, we utilized RNA-seq data obtained from two sets of mouse embryonic fibroblasts (MEFs): wild-type (WT) cells with basal mTOR activity and *Tsc1*^-/-^ cells with hyperactivated mTOR^10^. We calculated the TR for various genes and identified significant genes by applying specific criteria (chi-square *t* test *p* < 0.05 and TR difference >0.1). These significant genes are color-coded, with blue representing low mTOR activity and red indicating high mTOR activity (Fig. 1b). Interestingly, in contrast to the observed increase in 3’-UTR shortening APA events in the *Tsc1*^*-/-*^ transcriptome^10^, we observed distinct intronic APA events in both the WT and Tsc1-/- transcriptomes. Specifically, there were 188 events in WT and 253 events in *Tsc1*^*-/-*^ MEFs (as shown in Fig. 1b and Supplementary Table 1). This dichotomous pattern of intronic APA events was similar between *Tsc1*^*-/-*^ cells treated with Torin 1 (low mTOR activity) and mock-treated cells (high mTOR activity), with 251 events in the Torin 1 group and 90 events in the mock treatment group (Supplementary Table 2 and Supplementary Fig. 1a).

To investigate the consistency of the dichotomous intronic APA pattern across species, we conducted similar experiments in human breast cancer cell lines MCF7, BT549, and MDA-MB-361. Intriguingly, we observed a consistent intronic APA pattern in all the tested breast cancer cell lines (Fig. 1b and Supplementary Table 3). Specific examples of RNA-seq read alignments and real-time quantitative polymerase chain reaction (RT‒qPCR) analyses also confirmed the presence of dichotomous intronic APA events in both high- and low-mTOR environments (Fig. 1c−e and Supplementary Fig. 1b, c). To validate the role of mTOR, we performed RNAi-mediated knockdown of the mTOR gene in *Tsc1*^-/-^ MEFs, resulting in a replicated intronic APA pattern similar to that observed with pharmacological inhibition of mTOR activity (Supplementary Fig. 1d). Moreover, the datasets obtained from the three breast cancer cell lines revealed unique intronic APA events, with some overlap observed among the different cellular models (Supplementary Fig. 1e). Finally, when we conducted KEGG pathway analyses using the gene list of intronic APA events, we identified both unique and overlapping enrichment of biological pathways (Supplementary Fig. 1f). These findings suggest that intronic APA events are dynamic and have the potential to shed light on specific and common biological pathways in various cellular and biological models.

To determine the translational implications of intronic APA transcripts, we conducted western blot experiments, focusing on AGAP3 and SIN3B as representative examples. These proteins were chosen because intronic APA resulted in significant alterations in their protein lengths, and the antibodies were specific to the N-terminal regions of these proteins, enabling us to distinguish between full-length and truncated forms. Consistent with our RNA-seq data, our western blot results showed increased expression of C-terminal truncated AGAP3 and SIN3B proteins in *Tsc1*^-/-^ cells compared to WT MEFs, whereas the levels of full-length proteins remained unchanged (Fig. 1f). Intronic APA introduces “exonized” intron sequences into the 3’-most exon of the transcribed gene, which has the potential to give rise to C-terminal peptide sequences derived from the open reading frame through these exonized intron sequences (Supplementary Fig. 1g). Indeed, many of these translated peptide sequences from intronic APA transcript isoforms are documented in the RefSeq database^24^.

To validate the presence of these peptides in the proteome, we employed selected reaction monitoring (SRM), a targeted peptide detection method based on liquid chromatography-tandem mass spectrometry (LC‒MS/MS) (Supplementary Fig. 1h). Using this approach, we tentatively identified some of these peptide sequences (76 peptides in 40 proteins) in the WT and *Tsc1*^*-/-*^ cell extracts. This identification was further supported by synthesized peptides, with 10 out of 41 peptide sequences validated (Fig. 1g and Supplementary Fig. 1i). In summary, our findings suggest that intronic APA plays a role in shaping the C-terminome characteristics of the mTOR-regulated proteome.

### Pan-Cancer data analyses reveal distinct tumor- and normal tissue-enriched intronic APA profiles

**Fig. 2:**
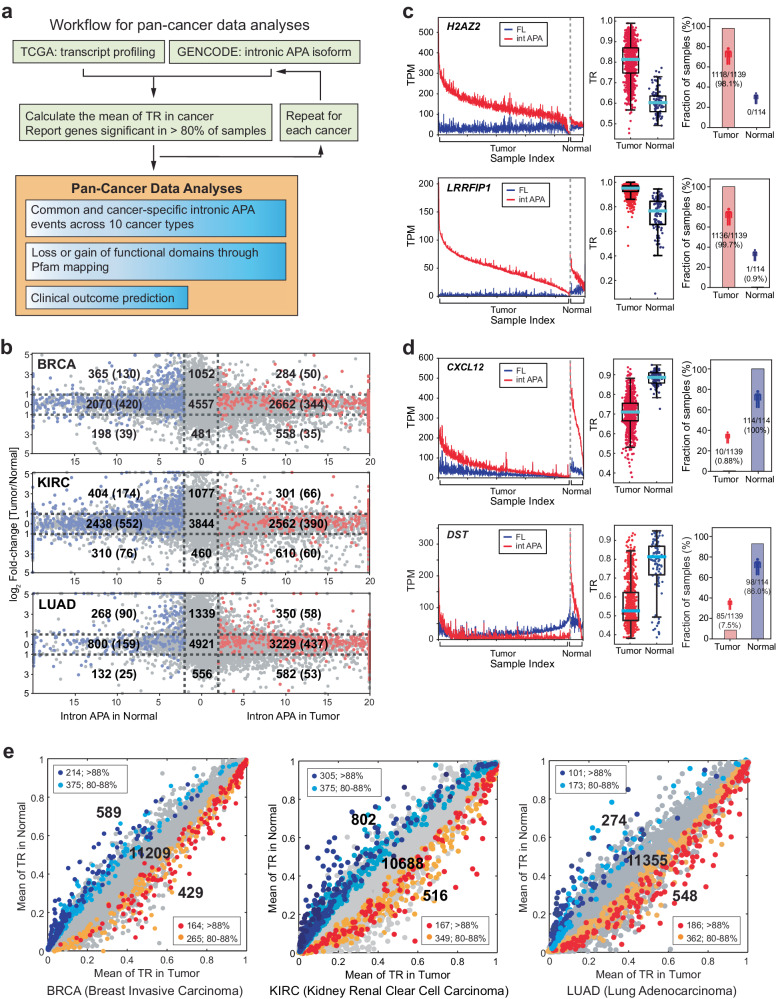

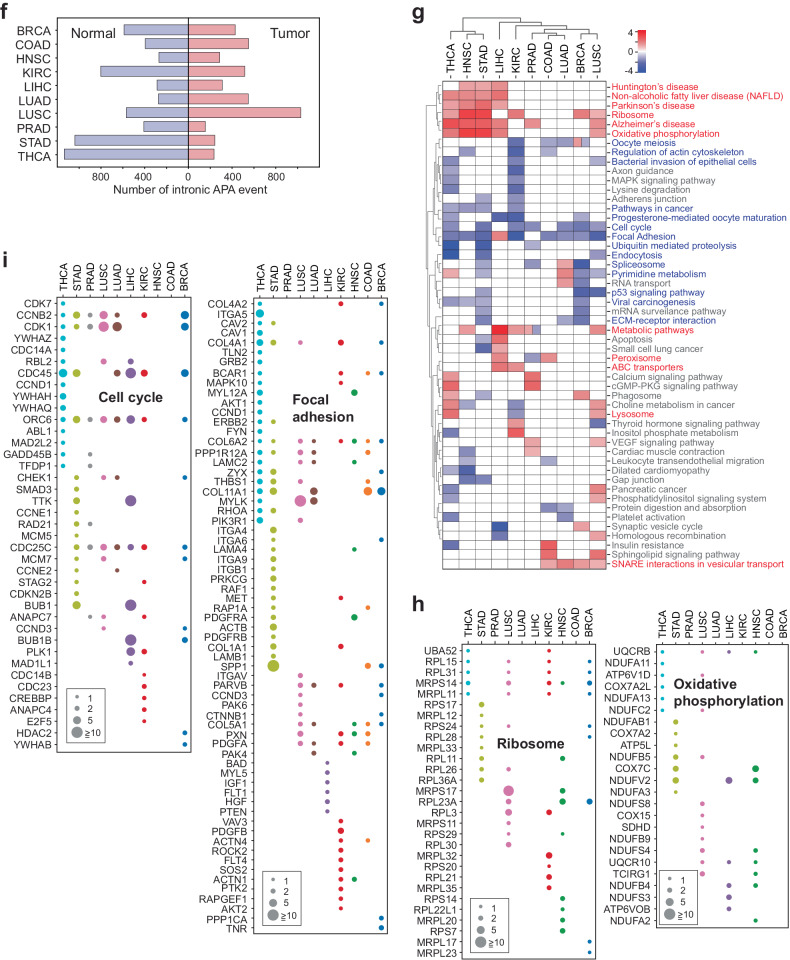
Discrete profile of intronic APA events in TCGA data. **a** A workflow depicting the pan-cancer data analyses for intronic APA events. **b** Differential expression analyses of genes with annotated intronic APA events in normal tissues and tumor samples. Three representative TCGA datasets (TCGA-BRCA (breast invasive carcinoma), TCGA-KIRC (kidney renal clear cell carcinoma), and TCGA-LUAD (lung adenocarcinoma)) are shown. The x-axis shows the significance of intronic APA events calculated using −log_10_(*p* value), whereas the y-axis shows the fold changes in gene expression in tumors compared with normal samples. Red dots indicate the genes showing significant intronic APA events conserved in 80% or more of tumor samples (i.e., 80% or more of tumor samples have higher TRs than the mean TR of normal tissue samples). The blue dots indicate the genes showing significant APA events for which 80% or more of the tumor samples had TRs lower than the mean TR of the normal tissue samples. **c** *H2AZ2* and *LRRFIP1* genes showing intronic APA events in BRCA tumors are presented as examples. The left panel displays the expression profiles of intronic APA (int APA; red) and full-length (FL; blue) transcripts across tumor samples and normal tissues. The middle panel presents the TRs of the corresponding genes in an individual sample using a box plot. The cyan bar indicates the median TR in tumor and normal samples. The right panel shows the distribution of *H2AZ2* and *LRRFIP1* TRs across the samples. The fraction of samples indicates the percentage of tumors that display a higher TR compared to the median of TR in the normal samples or vice versa. **d** *CXCL12* and *DST* genes showing intronic APA events in normal tissues in BRCA are presented as examples. Each panel shows the same data analyses as described in (**c**). **e** Scatter plots for intronic APA events in BRCA, KIRC, and LUAD. The TR means for genes with significant intronic APA events are color-coded. Genes showing intronic APA events in more than 88% of the samples are color-coded as blue (normal) or red (tumor). Genes with intronic APA events in 80−88% of the samples are shown in cyan (normal) or orange (tumor). **f** Overall distribution of intronic APA events in the TCGA pan-cancer cohort and corresponding normal tissue data. **g** Heatmap of the KEGG pathways enriched in intronic APA events across 10 cancer types. The KEGG pathways that are common to two or more cancers are displayed. The color scale represents −log_10_(*p* value) for pathways enriched in tumors and log_10_(*p* value) for pathways enriched in normal tissues: red-colored KEGG pathways are enriched in tumor samples, and blue-colored KEGG pathways are enriched in normal tissues. **h** Significant intronic APA genes associated with the ribosome or oxidative phosphorylation pathway in tumor samples according to the pan-cancer data. The scale of the circle indicates [the TR mean in tumors]/[the TR mean in normal tissues]. **i** Significant intronic APA genes associated with the cell cycle or focal adhesion pathways in normal tissues according to the pan-cancer data. The scale of the circle indicates [the TR mean in normal tissues]/[the TR mean in tumors].

Given the frequent dysregulation of mTOR in various cancers^21–24^, we hypothesized that the distinctive intronic APA pattern might also manifest in TCGA datasets. To delve into intronic APA profiles within TCGA datasets, we established a workflow aimed at examining intronic APA events and their clinical relevance across ten different cancer types, employing RNA-seq data from TCGA (Fig. 2a). We scrutinized transcript expression profiles from a comprehensive pool of 6099 samples encompassing both tumor and normal tissues across these diverse cancer types (Supplementary Fig. 2a). Through a comparative analysis of the expression profiles of intronic APA isoforms and full-length transcripts utilizing the GENCODE.v23 annotation, we identified significant intronic APA events (with a stringent *p* value threshold of <0.01) and illustrated them alongside their expression levels (Fig. 2b and Supplementary Fig. 2b). Using breast invasive carcinoma (BRCA) as a representative example, we identified significant intronic APA events in 3504 genes among 1061 tumor samples and 2635 genes among 109 normal samples (Fig. 2b). Our observations aligned with the findings from our prior cell line experiments (Fig. 1b) and provided evidence that widespread intronic APA events were present in both tumor and normal tissues across all analyzed cancer types (Fig. 2b and Supplementary Fig. 2b). Importantly, a substantial portion of these intronic APA events (e.g., 2070 out of 2633 cases in normal tissues and 2662 out of 3504 cases in BRCA tumors) did not exhibit differential gene expression. This observation underscores the absence of a correlation between differential gene expression and intronic APA events, underlining the limitations of conventional differential gene expression analyses in identifying intronic APA events.

The intronic APA events depicted in Fig. 2b and Supplementary Fig. 2b represent significant events observed across all samples, including individual variations. However, specific genes within the BRCA dataset such as *H2AZ2* (H2A histone family member V) and *LRRFIP1* (leucine-rich repeat flightless-interacting protein 1) exhibited a significant increase in TR in most tumor samples compared to normal tissues (Fig. 2c and Supplementary Fig. 2c). In contrast, the expression of genes such as *CXCL12* (C-X-C motif chemokine ligand 2) and *DST* (dystonin) exhibited an increase in TR in most normal tissues compared to tumor samples (Fig. 2d and Supplementary Fig. 2d). These examples illustrate that certain genes display intronic APA events consistently across both BRCA tumor samples and normal tissue collections. Furthermore, we identified conserved intronic APA events enriched in 80% or more of the tumors and normal tissues for each cancer type (Fig. 2b, e, Supplementary Fig. 2e, and Supplementary Table 4).

Our analysis of pan-cancer data revealed a prevailing pattern of discrete intronic APA profiles in both tumor and normal tissues across all investigated cancer types (Fig. 2f and Supplementary Table 4). We further examined the KEGG pathways associated with these APA profiles in each cancer type and generated a heatmap of the enriched pathways across the ten cancer types. Significantly, certain KEGG pathways, such as ribosome and oxidative phosphorylation, were consistently enriched in tumor samples of five or more cancer types, whereas KEGG pathways, such as cell cycle and focal adhesion, were commonly enriched in five or more normal tissue types (Fig. 2g). Additionally, we observed that some enriched KEGG pathways were unique to the tumor and normal tissues of each cancer type (Supplementary Fig. 2f). Intriguingly, although these KEGG pathways displayed conservation across different cancer types, the genes associated with these pathways varied in each cancer. For instance, the ribosome pathway was enriched by intronic APA events in six different cancer types, yet the profile of ribosomal genes differed among these cancer types (Fig. 2h). Similar observations were made for other KEGG pathways enriched by intronic APA events, including the cell cycle, oxidative phosphorylation, and focal adhesion (Fig. 2h, i). In summary, these findings suggest that mRNA truncation through intronic APA is a common characteristic observed in both cancer and normal tissue transcriptomes. This observation implies the potential significance of intronic APA events in targeting shared cellular pathways that may have relevance in cancer biology.

### Discrete intronic APA profiles reveal dynamic reorganization of protein functional domains in cancer proteomes

**Fig. 3:**
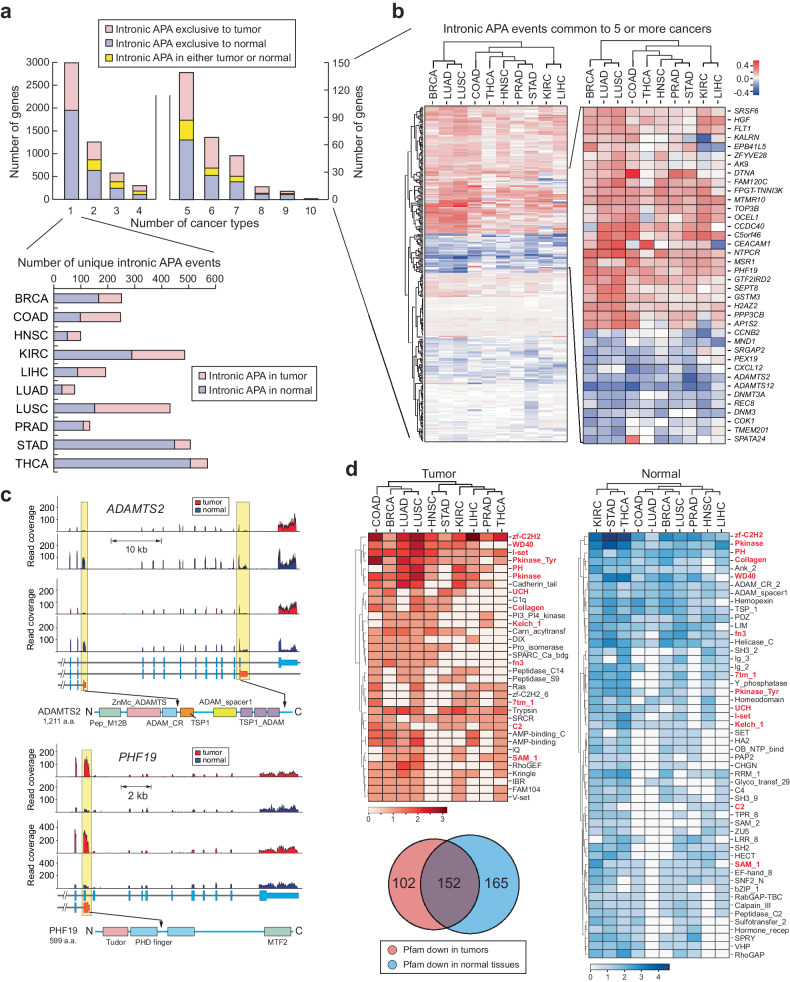

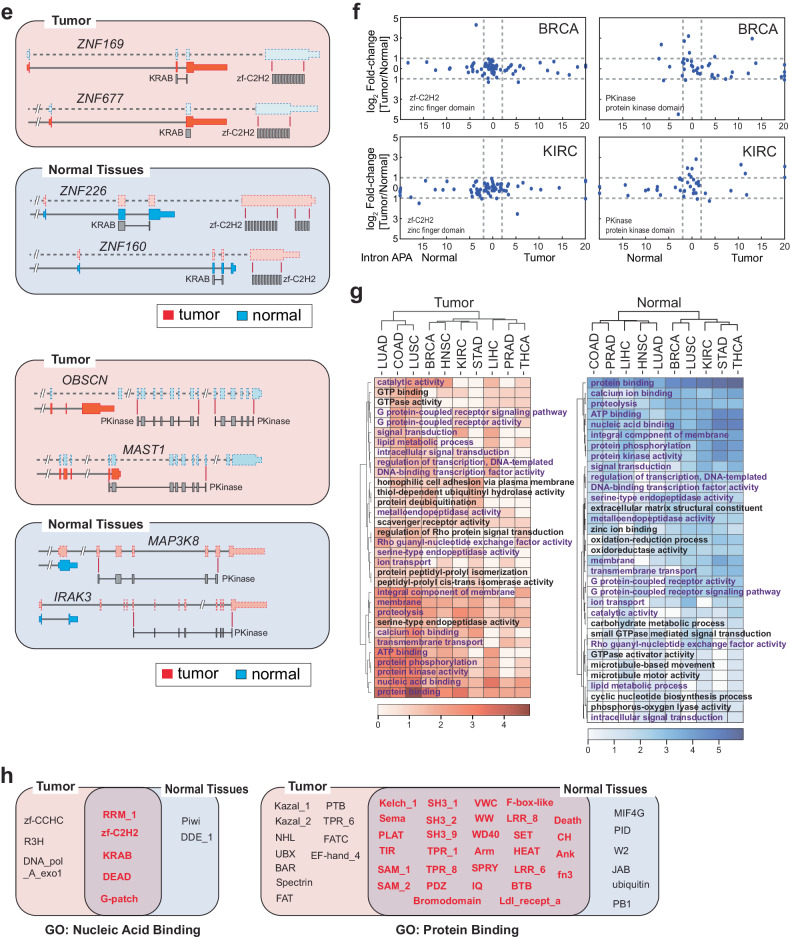
Intronic APA events in pan-cancer data and their associated molecular characteristics. **a** Frequency profile of intronic APA events in TCGA data. The number of intronic APA events is displayed by the occurrence in each cancer. **b** Heatmap of collective genes displaying a significant intronic APA event enriched in either tumor or normal samples across five or more cancer types. The color scale represents the differences in the mean TR between tumor and normal tissues. Select genes are highlighted in a zoomed-in heatmap as an example. Red and blue represent significant intronic APA events in tumors and normal tissues, respectively. **c** Two examples of intronic APA events from BRCA TCGA data. RNA-seq read alignments of two matched tumor and normal pairs are shown along with a schematic of the registered Pfam domains. Yellow boxes highlight the locations of intronic APA events. **d** Registered Pfam domains lost in tumors or normal tissues due to enrichment of intronic APA events. A Venn diagram showing the overall distribution of Pfam domains affected by intronic APA in the pan-cancer dataset. The heatmaps show the list of representative Pfam domains lost in tumors (red) or normal tissues (blue). The Pfam domains in the red text represent overlapping domains between tumors and normal tissues. The scale of the heatmap was calculated as follows: log_2_[# of APA events + 1]. Significant intronic APA events with domain changes in the exclusive exons of the full-length transcripts were considered. **e** Schematic representation of Pfam domain swapping in tumors and normal tissues by intronic APA events. Zinc finger domain (zf-C2H2) proteins and serine/threonine protein kinase (PKinase) proteins are shown as examples. **f** Differential gene expression for genes showing zf-C2H2 and PKinase domain swapping in BRCA and KIRC. The x-axis presents the significance of intronic APA events calculated as −log_10_(*p* value). **g** Heatmap of pan-cancer GO terms enriched in Pfam domains lost in normal tissues and tumor samples. The scale of the heatmap was calculated as log_2_[# of APA events + 1]. The GO terms common to both normal tissues and tumor samples are highlighted in violet font. **h** A representation of common and exclusive Pfam domains in cancer and normal tissues. Pfam domains associated with nucleic acid binding and protein binding are shown.

In our comprehensive analysis of cancer transcriptomes, we observed a substantial number of genes displaying intronic APA events. To investigate whether these events carry unique molecular signatures in cancer transcriptomes, we compiled a dataset encompassing 5400 significant intronic APA events occurring in more than 80% of normal tissues or tumors for each cancer type (Fig. 2e, Supplementary Fig. 2e, and Supplementary Table 4). Among these events, 2991 were specific to individual cancer types, whereas 2409 were common across two or more cancer types (Fig. 3a). Notably, more than 10% of these shared events (279 intronic APA events) were conserved in five or more cancer types. Among these conserved events, 37 were found in either tumor or normal tissues, 118 were exclusive to tumors, and 124 were exclusive to normal tissues (Fig. 3a). Furthermore, we identified unique intronic APA events specific to each cancer type (Supplementary Fig. 3a).

Our analysis highlighted *ADAMTS2* (a disintegrin-like and metalloprotease with thrombospondin type 1 motif 2) as one of the most consistently conserved intronic APA events across all 10 normal tissues, suggesting that the truncated *ADAMTS2* transcripts (ENST00000274609.5 and ENST00000518335.3) are more abundant in normal tissues than in tumor tissues (Fig. 3b, c). Conversely, *PHF19* (PHD finger protein 19) exhibited increased expression of the intronic APA transcript (ENST00000312189.10) in tumor samples from nine different cancer types (Fig. 3b, c). These intronic APA events in genes such as *ADAMTS2* and *PHF19* generate C-terminal truncated proteins. For example, *ADAMTS2* produces two truncated isoforms, one (ENST00000274609.5) of which lacks functional domains like ADAM_spacer1, TSP1, and TSP1_ADAM (thrombospondin type 1 domain), which are found in the full-length ADAMTS2 protein (Fig. 3c). Similarly, the tumor-enriched PHF19 intronic APA transcript generated a truncated PHF19 protein lacking one PHD finger domain and the Mtf2_C (polycomb-like MTF2 factor 2) domain (Fig. 3c). This finding indicates that widespread intronic APA events across the transcriptome can result in a broad-scale reduction in protein functional domains. This reduction may lead to the loss of conventional protein functions. However, it also opens the possibility of gaining new functions through the remaining functional domains in the truncated proteins.

To explore the global changes in protein functional domains associated with our pan-cancer analysis, we mapped the missing mRNA regions due to intronic APA events to the Pfam database for functional domain identification^26^. Our analysis revealed that many Pfam domains were absent in both tumors (254 domains) and normal tissues (317 domains) (Fig. 3d), with 34 and 52 domains, respectively, being common to five or more cancer types (the heatmap in Fig. 3d and Supplementary Table 5). Surprisingly, a significant portion of these missing Pfam domains (152 domains) overlapped between tumors and normal tissues, many of which were common to five or more cancer types (highlighted in the red font in the heatmap of Fig. 3d).

One notable functional domain that is missing across all proteomes in both tumor and normal samples is the Cys2His2-type zinc finger domain (zf-C2H2). In this case, a group of zinc finger protein (ZNF) genes within tumors and normal tissues lost the zf-C2H2 domain, retaining only the Krüppel-associated box (KRAB) domain (Fig. 3e). Importantly, most ZNF genes identified in this analysis did not exhibit differential expression between tumor and normal tissue samples. This finding suggests that traditional differential gene expression analyses are insufficient for identifying such functional loss of ZNFs in cancer and normal tissue proteomes (Fig. 3f). These findings also imply that this zf-C2H2 domain-containing protein family employs intronic APA to reconfigure the inherited functionality of zinc finger proteins in both tumor and normal tissues without altering their expression levels.

Similarly, multiple Pfam domains, such as protein kinases (PKinase) and tryptophan-aspartate repeats (WD40), exhibited comparable restructuring of functional domains in both tumor and normal tissue samples (Fig. 3e, f and Supplementary Fig. 3b). To further investigate the association between this intronic APA-driven domainomics atlas and biological functions, we employed Gene Ontology (GO) analysis as a reference. Similar to our findings in the Pfam domain analysis, we discovered that numerous GO terms linked to the affected Pfam domains are common to both tumor and normal tissue samples (Fig. 3g and Supplementary Table 6).

For instance, the GO term related to nucleic acid binding was enriched by Pfams such as RNA recognition motif 1 (RRM_1), zf-C2H2, DEAD (DEAD/DEAH box helicase), and Krüppel-associated box (KRAB) (Fig. 3h). This finding suggested that genome-wide transcriptional regulation might also be orchestrated by the intronic APA-driven reorganization of KRAB domains in both cancer and normal tissue proteomes. Nevertheless, there are Pfam domains specific to the nucleic acid binding GO term in either tumors (zf-CCHC (zinc knuckle) and R3H) or normal tissues (Piwi and DDE_1 (endonuclease)). This highlights Pfam domains unique to either tumor or normal tissue that represent distinct molecular pathways in pathological and nonpathological states (Fig. 3h). A similar pattern of Pfam domain redistribution was observed for the protein binding GO term (Fig. 3h). In summary, these results reveal an unexpected finding of intronic APA-driven domainomics that influences the regulation of biological functions through proteome-wide restructuring of functional domains. Importantly, this reorganization occurs independently of differential gene expression.

### Intronic APA patterns are linked to clinical variables

In prior investigations of pan-cancer data, researchers identified signatures that were common to various cancers as well as those specific to individual cancer types. These findings were based on differential gene expression and alternative polyadenylation (APA) events occurring in the 3’-UTR^27,28^. Our analysis of intronic APA data within the TCGA dataset revealed distinctive molecular characteristics associated with the reprogramming of protein functional domains and the regulation of biological pathways through intronic APA. Notably, we observed no significant correlation between differential gene expression and intronic APA across either tumor or normal tissue samples (Fig. 2b and Supplementary Fig. 2b). This led us to explore whether the molecular signatures derived from intronic APA events could be linked to clinical parameters. Specifically, we focused on genes exhibiting significant differences in the TR and assessed their clinical relevance, particularly concerning hormone receptor phenotypes (ER, HER2, PR, and triple negative) in breast cancer.

**Fig. 4:**
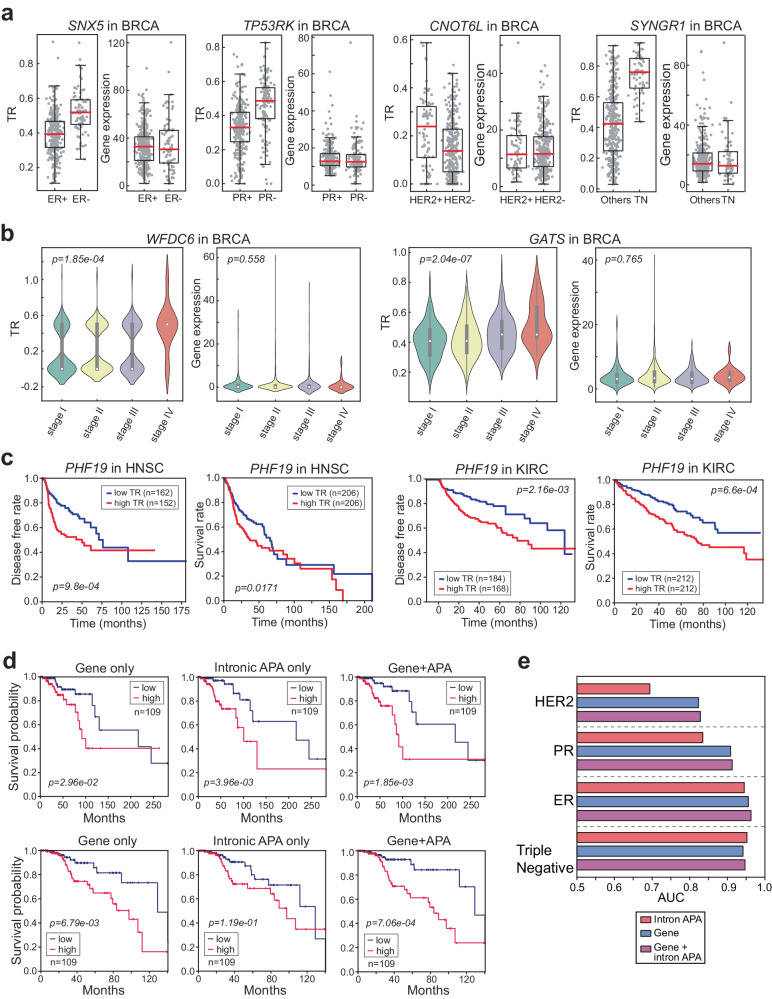
Intronic APA is associated with clinical variables. **a** Boxplots present four examples where intronic APA events are associated with hormone receptor phenotypes but not with the corresponding gene expression levels in BRCA data. The significance of these associations was assessed using unpaired t tests, and the *p* values were as follows: *SNX5* TR *p* = 6.24e-18, gene expression (GE) *p* = 0.529; *TP53RK* TR *p* = 1.02e-16, GE *p* = 0.851; *CNOT6L* TR *p* = 4.65e-6, GE *p* = 0.922; *SYNGR1* TR *p* = 1.05e-27, GE *p* = 0.701. **b** Violin plots illustrating two exemplary genes demonstrating significant intronic APA events but not significant differential gene expression in cancer stages. **c** Kaplan‒Meier (KM) plots illustrating the correlation between the TR of *PHF19* and the disease-free rate or survival rate of HNSC and KIRC patients. **d** KM plots for the high- (red line) and low-risk (blue line) groups generated based on gene expression, TR of intronic APA events, and the combination of gene expression and TR. The upper three KM plots represent case 1, and the lower three KM plots represent case 2. The *p* values were determined using the log-rank test. **e** Prediction results of hormone receptor phenotypes based on gene expression, TR of intronic APA events, and the combination of gene expression and TR. SVM was used for the prediction task, and the mean AUC of 100 repeats (i.e., splitting of trainings, validation, and test sets) is reported.

We observed that the TRs of certain genes, such as *SNX5* and *NGEF*, strongly correlated with the estrogen receptor (ER) phenotype in breast cancer. This association was particularly notable when differential gene expression failed to distinguish between tumor samples based on hormone receptor phenotypes (as shown in Fig. 4a and Supplementary Fig. 4a). Similarly, the TRs of genes such as *TP53RK* and *CNOT6L* were linked to progesterone (PR) and human epidermal growth factor (HER2) receptor phenotypes, respectively (Fig. 4a). Notably, the TRs of several genes, including *SYNGR1*, *GTF2IRD2*, and *FAM120C*, exhibited increased levels in the tumor samples of triple-negative breast cancer patients, whereas genes such as *TVP23C*, *ICAM3*, and *RIMKLB* displayed decreased TRs in triple-negative breast cancer tumor samples (Fig. 4a and Supplementary Fig. 4a).

Furthermore, we found that the TRs of genes was associated with disease stage in various cancer types (Fig. 4b and Supplementary Fig. 4b). Overall, there was an increase in the TR (e.g., *WFDC6*, *GATS*, *FMNL3*, *COQ4*, and *MEOIC*) as the cancer stage progressed, whereas a decrease in the TR was noted for *PARVA* (Fig. 4b and Supplementary Fig. 4b). Notably, the differential expression of these genes did not demonstrate any significant associations with these clinical parameters. These results indicate that the TRs of genes are linked to disease phenotypes and unveil previously unnoticed molecular signatures.

In light of these findings, we conducted further investigations into the correlation between TR and long-term clinical outcomes, such as disease-free survival time and survival rate. Our analyses revealed that certain genes, such as *PHF19* and *VRK3*, which are truncated in 9 and 5 different cancers, respectively, exhibit a negative correlation between the TR and disease-free survival time or survival rate in specific cancer types (Fig. 4c and Supplementary Fig. 4c). To evaluate the prognostic significance of intronic APA events and gene expression profiles, we employed a Cox proportional hazards model with an elastic net penalty^29^. Initially, we utilized only the TR values of intronic APA events or gene expression profiles as molecular covariates to generate low- and high-risk patient groups. We then assessed the significance of our findings using Kaplan‒Meier (KM) plots and the log-rank test. In case 1 (the first row in Fig. 4d), intronic APA exhibited superior prognostic power compared to gene expression alone in predicting patient survival probability (Fig. 4d). Conversely, in case 2 (the second row in Fig. 4d), the performance of intronic APA was weaker than that of gene expression (Fig. 4d). Given the variable performance of intronic APA as a standalone variable, we asked whether intronic APA events combined with gene expression covariates exhibit an improved ability to predict survival. In both cases where intronic APA events showed mixed performance compared to gene expression, the combination of intronic APA and gene expression significantly improved the ability to predict patient survival compared to the prediction generated using either covariates of intronic APA events or the gene expression signatures alone (Fig. 4d).

We also explored the potential of enhancing clinical outcome predictions in breast cancer by integrating intronic APA data into gene expression analysis. To do this, we applied a conventional classification method, support vector machine (SVM), to investigate the role of intronic APA events as a molecular signature in predicting clinical outcomes in breast cancer patients. Our analyses revealed that when gene expression and intronic APA were integrated, they outperformed gene expression or intronic APA alone, particularly in predicting outcomes related to estrogen receptor (ER), human epidermal growth factor receptor 2 (HER2), and progesterone receptor (PR), as indicated by the higher area under the curve (AUC) score. Remarkably, in regard to triple-negative breast cancer, the performance of the integrated transcriptomic signatures was superior to that of gene expression alone, with intronic APA showing the most significant improvement in prediction performance (Fig. 4e). In summary, our findings suggest that intronic APA events can function as a valuable component of clinical signatures, offering a substantial contribution to the prediction of survival and cancer outcomes. This contribution goes beyond the capabilities of commonly used genomic data analyses, such as gene expression alone.

### Intronic APA highlights the importance of C-terminomics in the cancer proteome

Traditionally, intronic APA has been viewed as a process leading to the premature termination of transcription, resulting in the production of truncated mRNA molecules that can subsequently impact the length of proteins^30^. This phenomenon has subsequent implications for domainomics, potentially affecting the entire proteome. Our data, illustrated in Fig. 3c, d, indicate that intronic APA can cause significant alterations in domainomics in both tumor and normal tissues. However, it is important to note that not all cases of intronic APA result in substantial changes in protein length, as exemplified by the case of ADAMTS2 (the second transcript isoform, ENST00000518335.3), which maintains all Pfam domains despite intronic APA (as shown in Fig. 3c).

**Fig. 5:**
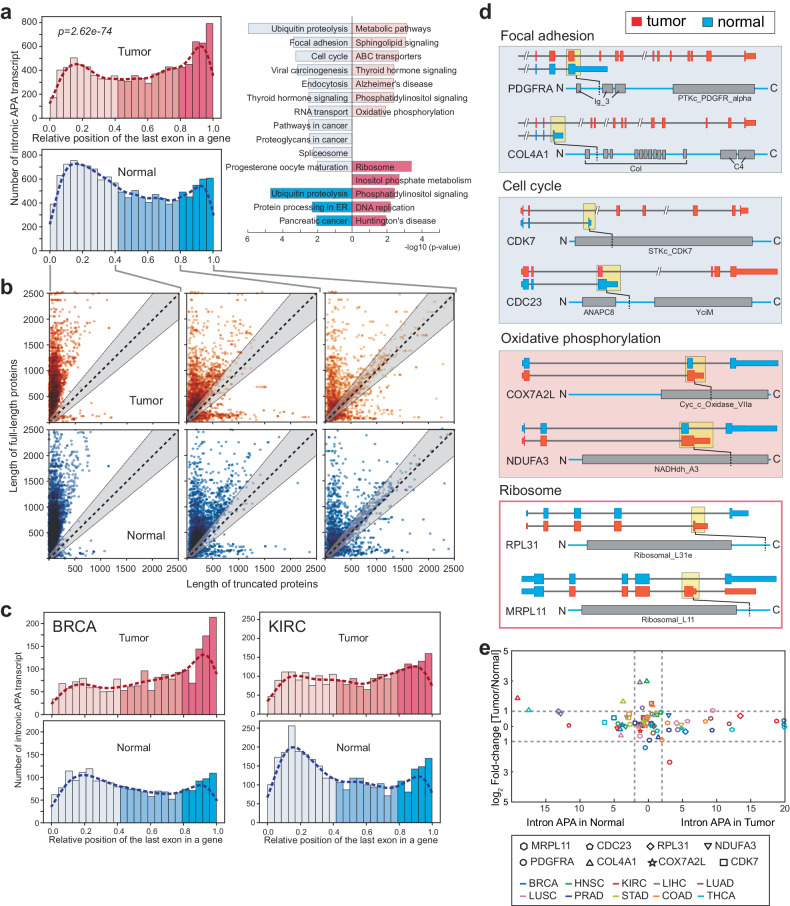
Intronic APA renders diverse C-terminome features. **a** The histogram displays the distribution of relative positions of the 3’-last exon of intronic APA transcripts to the full-length transcript in the same gene in the pan-cancer dataset. Enriched KEGG pathways corresponding to early or late termination of transcripts are presented. **b** Comparison of full-length vs. truncated proteins grouped by the relative position of intronic APA events. The gray shaded area denotes the length of truncated proteins that fall within ±10% of the corresponding full-length proteins. **c** The histogram shows the distribution of intronic APA positions in BRCA and KIRC. **d** A schematic representation of Pfam domains that are lost by intronic APA and enriched in select KEGG pathways. Yellow boxes where the open reading frames of intronic APA transcripts end are linked to the positions of the truncated proteins in the diagram. **e** Differential gene expression for select genes enriched in the KEGG pathways shown in (**a**). The selected genes are color-coded by cancer type where intronic APA events are statistically significant.

To gain insights into how transcriptome-wide intronic APA events influence protein length within both the cancer and normal tissue proteomes, we examined the relative position of the last exon containing the termination codon in truncated transcripts compared to fully annotated full-length transcripts using data from various cancers and normal tissues. The overall distribution of these last exons, stemming from transcript truncation, exhibited a bimodal pattern in the current genome annotation (Supplementary Fig. 5a). Notably, this bimodal distribution of intronic APA events was consistent across both tumor and normal tissue data analyses. However, the enrichment of APA events varied significantly between the two pathological conditions. In normal tissues, intronic APA events tend to cause earlier termination of transcription (up to 40% of the length of full-length transcripts); however, in tumors, termination due to intronic APA yields transcripts that closely resemble that of full-length transcripts (Fig. 5a and Supplementary Table 7).

We conducted KEGG pathway analysis on intronic APA events belonging to each group, classified by the relative length of transcriptional termination in tumors and normal tissues. This analysis highlighted specific biological pathways. Interestingly, the focal adhesion and cell cycle pathways were enriched in the group of intronic APA events occurring in earlier exons in normal tissues, whereas the oxidative phosphorylation pathway exhibited enrichment in the same group characterized by earlier terminations in tumors. Conversely, the ribosome pathway was enriched in the group of intronic APA events occurring in later exons (beyond 80% of transcript length) in tumors (Fig. 5a and Supplementary Table 7).

Although earlier termination of transcription results in truncated mRNAs, these truncated mRNAs contain the essential elements required for translation into proteins. To understand how these uneven distributions of terminal exons affect protein length in the cancer proteome, we calculated the lengths of the truncated and corresponding full-length proteins and classified them into three different groups based on the relative ranges of truncation positions: 0.0−0.4, 0.4−0.8, and 0.8−1.0. The scatter plots depicting protein lengths show that intronic APA events causing truncation of up to 40% of mRNA length mostly yield significantly truncated proteins in both tumors and normal tissues (as observed in the left plots of Fig. 5b). As the relative position of intronic APA events extends into the range of 0.4−0.8 truncation, the trend of producing substantially truncated proteins gradually diminishes, and longer proteins start to appear (middle plots in Fig. 5b). Intronic APA events that lead to similar mRNA lengths (relative truncation position ranges between 0.8 and 1.0) do not significantly affect protein length; many truncated proteins fall within the gray area, indicating ±20% variation in the length of corresponding full-length proteins (right plot in Fig. 5b). It is also worth noting that numerous genes produce much longer proteins due to intronic APA events, regardless of the relative truncation position (as evident in the bottom half area below the diagonal gray region in Fig. 5b). Further examination of these findings in individual cancer types confirmed the presence of similar disproportionate distributions of relative intronic APA positions between tumors and normal tissues (Fig. 5c and Supplementary Fig. 5b).

We also noticed that specific conserved KEGG pathways, such as focal adhesion, cell cycle, and ribosome, which were highlighted in pan-cancer and normal tissue intronic APA events (as shown in Fig. 2g−i), also appeared in the data analysis related to early or late-terminating intronic APA events in tumors and normal tissues (Fig. 5a). Given this overlap, we explored whether genes within these pathways demonstrated any connection between protein length and the reorganization of Pfam domains, which could impact protein function. Intriguingly, genes associated with focal adhesion and cell cycle pathways produced truncated proteins that lost their C-terminal functional domains due to intronic APA, suggesting potential compromises in the functionality of these proteins (Fig. 5d). Similar observations were made for genes linked to the oxidative phosphorylation pathway (Fig. 5d). However, in the case of genes associated with ribosomes, the C-terminal sequences were altered without affecting the arrangement of known functional domains (Fig. 5d).

Furthermore, most of the genes presented in Fig. 5d did not exhibit differential gene expression in the context of 10 different cancer types (Fig. 5e), which aligns with our previous observations (Fig. 3f). Consequently, our findings suggest that intronic APA is employed differently in tumor and normal tissue gene expression and in the regulation of biological pathways. In normal tissues, diverse biological pathways are influenced by an interruption of transcriptional elongation (Fig. 5a−d). In contrast, in tumors, intronic APA seems to primarily modify the C-terminal amino acid sequence by altering the choice of the terminal exon, resulting in proteins of similar length without significant alterations to their functional domains.

### JMJD6 C-terminal variants generated by intronic APA confer opposing functions

**Fig. 6:**
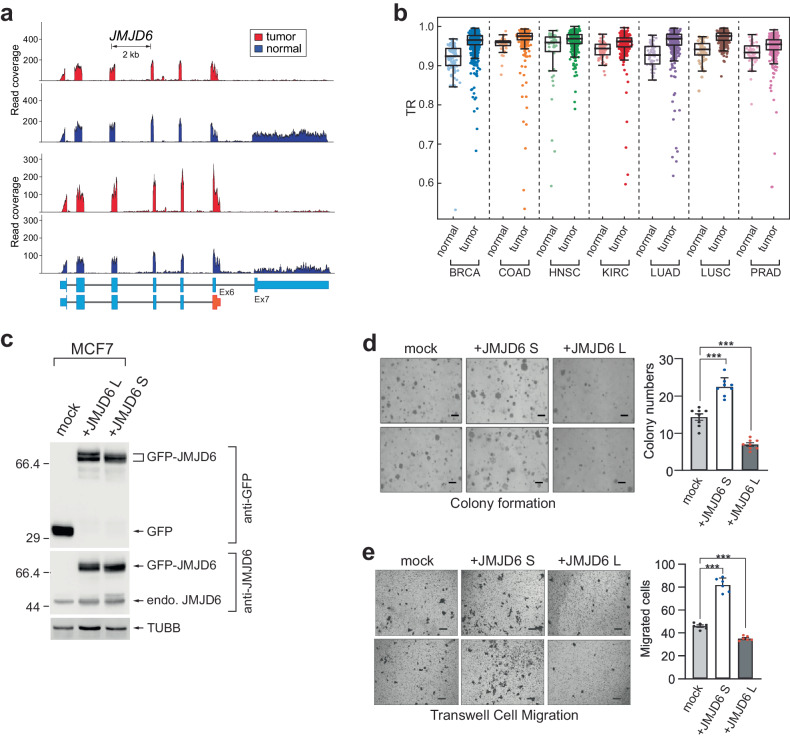
An intronic APA-driven JMJD6 C-terminal change is pathogenic. **a** Exemplary RNA-seq read alignments for *JMJD6* in BRCA data. Two pairs of patient tumor samples and corresponding normal tissue samples are shown. **b** TRs of *JMJD6* in 7 types of cancer. *p* values for each cancer type are as follows. BRCA: 7.435e-17, COAD: 2.017e-4, HNSC: 5.634e-3, KIRC: 1.383e-8, LUAD: 9.592e-11, LUSC: 9.482e-12, PRAD: 1.544e-4. **c** western blot analyses of JMJD6 overexpression in MCF7 breast cancer cells. GFP alone (mock), GFP-JMJD6 long, and GFP-JMJD6 short were overexpressed in MCF7 cells, and their effects on cancer cell behaviors were measured. Endogenous JMJD6 expression was also examined in the same cell extracts. Beta-tubulin was used as a loading control for the western blot analyses. Anchorage-independent growth assessed using soft agar assays (**d**) and migration assays (**e**) in mock, GFP-tagged JMJD6 long isoform, or GFP-tagged JMJD6 short isoform-expressing MCF-7 cells. Representative images are shown. The quantification of the images is shown in the bar graph. Scale bar = 100 nm. The data are shown as the mean (SD). **p < 0.01, two-tailed Student’s *t* test.

The findings presented in Fig. 5 raise an important question about the biological significance of C-terminal sequence exchanges through intronic APA events. The fact that such exchanged peptide sequences are often similar in length and possibly unstructured in 3-dimensional protein structure analyses may lead to the underestimation of their significance. However, our observations regarding *JMJD6* (jumonji domain-containing 6, arginine demethylase and lysine hydroxylase) indicate that intron 6 APA-driven short isoform expression is almost exclusively observed in tumor samples, whereas both long and short isoforms are expressed in normal tissues (Fig. 6a). Furthermore, our pan-cancer data analysis revealed that *JMJD6* exhibits a significant intronic APA event that is common to seven different cancer types (Fig. 6b). Notably, several studies have investigated the role of JMJD6 in cancer biology but have suggested contradictory models for its function in cancer pathogenesis^19,31–36^. We hypothesize that one reason for these contradictory findings is the different isoform usages in these studies. To test this idea and explore the functional differences between the two possible isoforms of JMJD6, we overexpressed short or long isoforms of GFP-tagged JMJD6 in the MCF7 breast cancer cell line and assessed their effects on colony formation and cell migration. Our results demonstrated that while overexpression of the JMJD6 long isoform impaired colony formation and migration, overexpression of the JMJD6 short isoform promoted colony formation and enhanced migration (Fig. 6c, d, e). These findings provide evidence that the two JMJD6 isoforms exhibit distinct functions in MCF7 cancer cells due to changes in amino acid sequences in their C-termini. Therefore, these results strongly suggest that alterations in the C-terminal amino acid sequences of the two isoforms result in the multifaceted functionality of the gene and highlight the potential role of the intron APA-driven C-terminome in the regulation of disease pathogenesis.

## Discussion

Polyadenylation of mRNA transcripts is a crucial process in gene expression^1,2,37^. Mapping technologies and various pipelines for cataloging polyadenylation sites have demonstrated that APA is a dynamic process regulated by diverse biological contexts^2,37–39^. APA in the 3’-UTR of mRNAs is associated with cancer pathogenesis and various cellular conditions, including mTOR signaling^10,40–42^. These studies have highlighted 3’-UTR APA as a process that shortens the 3’-UTR length of mRNAs in cancer and upregulated mTOR context^10,14,43^. However, this study revealed that intronic APA events occur in a dichotomous manner, featuring both upregulation and downregulation of intronic APA in the transcriptome (Figs. 1b,2b, e, f). These findings contrast with previous reports showing that APA is a unidirectional process that shortens transcripts in the transcriptome of a given biological or disease context^10,14,43^. Intronic APA is intricately linked with splicing processes, as the selection and strength of 3’-splice sites across various biological contexts and the presence of polyadenylation signals within introns influence intronic APA events. Consequently, alteration of the last exon through intronic APA is often considered a form of alternative splicing. Notably, our prior research demonstrated that mTOR plays a regulatory role in alternative splicing across the transcriptome^44^. This finding suggested that the association of mTOR with alternative splicing might be crucial for generating diverse profiles of intronic APAs. In contrast, APA in the 3’-UTR is predominantly governed by polyadenylation factors and consensus sequence motifs located both upstream and downstream of the polyadenylation site, including the polyadenylation signal itself. This fundamental mechanistic difference between intronic APA and 3’-UTR APA suggests that mTOR may contribute differently to the complexity and functionality of the proteome.

The human GENCODE^45^ annotation contains 20,114 coding genes, and greater than 50% of those genes (12,453 genes) contain at least one annotated intronic APA event. Therefore, the 5400 intronic APA events (43% of the total annotated intronic APA events and 27% of the total genes in the human genome) discovered in our pan-cancer and normal tissue data analyses emphasize the importance of intronic APA in understanding the cancer transcriptome. Most intronic APA events are not coupled to differential gene expression (Fig. 2b and Supplementary Fig. 2b), and the combination of intronic APA and differential gene expression analyses increases the prediction power in cancer research (Fig. 4d, e), confirming the necessity of performing comprehensive intronic APA profiling to understand the cancer transcriptome. Identifying common and unique intronic APA genes among different types of cancer is valuable for their future applications in translational and basic biomedical research.

Intronic APA transcripts in cancer and normal tissue transcriptomes might exhibit potential value as biomarkers and therapeutic targets in cancer pathogenesis^5^. However, the biological significance of intronic APA is largely elusive due to the lack of connections to the functional proteome. Several lines of evidence show that truncated proteins function differently and steer biology towards different directions^46–50^. Our in silico analyses of functional domains affected by intronic APAs in pan-cancer and normal tissue data revealed new multifaceted roles of intronic APAs in the transcriptome. Characterization of the gain or loss of functional domains due to dichotomous intronic APA profiles revealed a new mechanistic role of intronic APA in the reprogramming of functional domains in the same family of proteins. For instance, a subset of zinc finger proteins (zf-C2H2) that bind nucleic acids, especially DNA, and regulate transcription and other activities of the genome^51^ is truncated in normal tissues, whereas another subset of truncated zf-C2H2 proteins is expressed in tumors (Fig. 3e, g). At the proteome level, this phenomenon looks like turning on or off the function of zinc finger proteins by adding/deleting the zf-C2H2 domain to a subset of genes within the same family of proteins depending on the biological or disease context. Domain swaps with many specific functional domains, including those of PKinase and WD40 (Fig. 3d), suggest that various molecular functions and biological reactions are regulated in this manner. Surprisingly, most of the genes associated with domain swaps did not exhibit differential gene expression, further highlighting the regulatory role of intronic APA in multilayered gene expression pathways.

Further efforts to connect intronic APA to the functional proteome revealed that the relative positions of intronic APA events differ between normal tissues and tumors (Fig. 4a). Specifically, in normal tissues, earlier termination of transcription could interrupt the expression of genes critical for cell division and proliferation by truncating functional domains in the C-terminus. In contrast, in tumors, this earlier termination affects oxidative phosphorylation (Fig. 4a, d). Genes associated with pathways such as ribosomes showed intronic APA events close to the last exon of the full-length annotation, which resulted in the replacement of short peptide sequences (Fig. 4a, d). Thus, intronic APA not only truncates the expression of full-length proteins but also reprograms the C-terminome of the proteome by replacing C-terminal peptide sequences. These findings raise an important question in biology: can the proteome efficiency of cancers also be reprogrammed through C-terminomics? In this context, JMJD6 is an interesting example.

JMJD6 is an arginine demethylase and a lysine hydroxylase enzyme. Its overexpression is associated with poor patient survival^35,52^, and it has been shown to promote tumor growth, migration, and colony formation^32,33,53–56^. However, previous studies on the role of JMJD6 in cancer have reported conflicting results^31,57^. For example, Lee et al. suggested that JMJD6 overexpression leads to an increase in anchorage-independent growth in human oral squamous cell carcinoma, whereas Poulard et al. reported that JMJD6 overexpression reduces colony formation in MCF7 human breast cancer cells^31,57^. These conflicting findings led us to investigate the isoform-specific function of JMJD6 and whether previous studies overexpressed different isoforms of JMJD6. Here, we show that the short and long JMJD6 APA isoforms have opposing functions in cancer cell behaviors. We found that APA of *JMJD6* can result in a functional pivot between acting as an oncogenic protein and acting as a tumor suppressor. This finding is consistent with the expression profiles of JMJD6 APA isoforms in normal and tumor samples from cancer patients, suggesting that cancer cells maximize the expression of the short form of JMJD6, which functions as an oncogene. However, the mechanisms by which the short and long JMJD6 isoforms function differently remain unclear. Considering that the entire catalytic domain of JMJD6 is intact between the two isoforms, it is likely that the biochemical and/or biophysical characteristics of JMJD6 are regulated by this unstructured C-terminal domain sequence. Several studies have demonstrated that JMJD6 functions as a dimer and that the deletion of the C-terminal region in the longer isoform of JMJD6 leads to a loss of its catalytic activity^58,59^. Consequently, the additional C-terminal sequences present in the long form of JMJD6 are likely to confer distinct enzymatic properties compared to those of the short form. This notion suggests that the short amino acid sequences existing in the C-terminus of proteins between the isoforms, in addition to structural differences, significantly influence the enzyme’s functional characteristics, emphasizing the significance of the C-terminome.

In conclusion, we present evidence of a dichotomous intronic APA profile in both cellular and cancer contexts, which yields truncated mRNA transcripts that can still be translated into functional proteins. Our in silico analyses of intronic APA profiles revealed a previously unappreciated regulatory role of intronic APA in the functional proteome, specifically through its impact on C-terminomics. We found that intronic APAs can extensively exchange functional domains within the same family of proteins in their C-terminal regions and potentially alter the C-terminal structure of proteins by introducing new unstructured peptide sequences. These alterations have the potential to affect the inherent functions of proteins and create a novel regulatory layer in the proteome.

## Supplementary information

Supplementary materials

Supplementary Table 1

Supplementary Table 2

Supplementary Table 3

Supplementary Table 4

Supplementary Table 5

Supplementary Table 6

Supplementary Table 7
