# Supplementary material for: Dichotomous intronic polyadenylation profiles reveal multifaceted gene functions in the pan-cancer transcriptome
Source: Exp Mol Med. 2024 Oct 1;56(10):2145–61. doi: 10.1038/s12276-024-01289-w (PMC11541570; doi:10.1038/s12276-024-01289-w)
Supplement: Supplementary file 1 — Supplementary materials [file 12276_2024_1289_MOESM1_ESM.pdf]

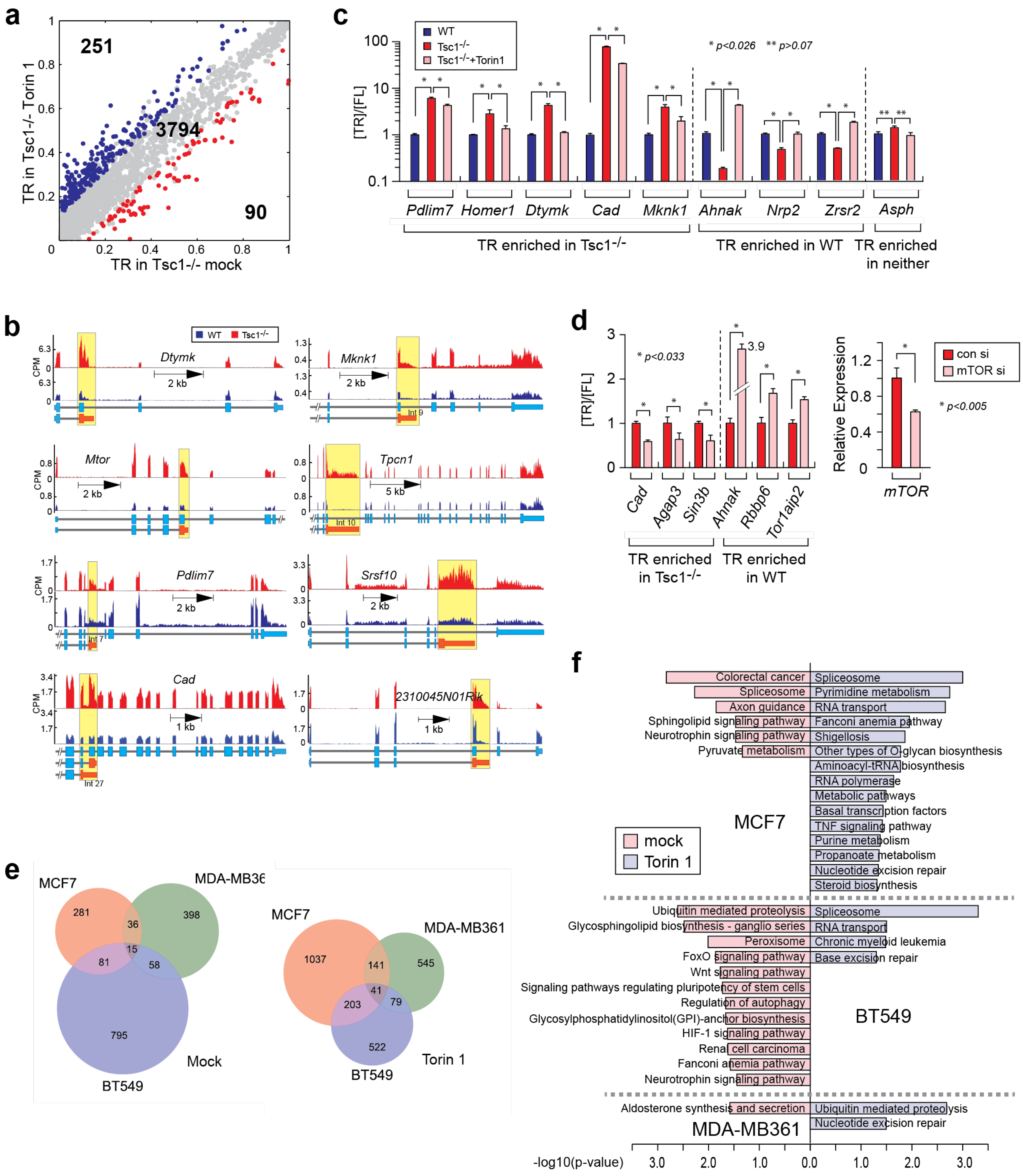
a
251
3794
90
TR in Tsc1-/- Torin 1
TR in Tsc1-/- mock
c
WT
Tsc1-/-
Tsc1-/-+Torin1
* p<0.026 ** p>0.07
[TR]/[FL]
Pdlim7
Homer1
Dtymk
Cad
Mknk1
Ahnak
Nrp2
Zrsr2
Asph
TR enriched in Tsc1-/-
TR enriched in WT
TR enriched in neither
b
WT
Tsc1-/-
CPM
Dtymk
Mknk1
Mtor
Tpcn1
Pdlim7
Srsf10
Cad
2310045N01Rik
2 kb
5 kb
1 kb
Int 9
Int 10
Int 7
Int 27
d
* p<0.033
[TR]/[FL]
3.9
Cad
Agap3
Sin3b
Ahnak
Rbbp6
Tor1aip2
TR enriched in Tsc1-/-
TR enriched in WT
Relative Expression
con si
mTOR si
* p<0.005
mTOR
e
MCF7
MDA-MB36
Mock
BT549
281
36
398
15
81
58
795
MCF7
MDA-MB361
Torin 1
BT549
1037
141
545
41
203
79
522
f
mock
Torin 1
MCF7
Colorectal cancer
Spliceosome
Axon guidance
Sphingolipid signaling pathway
Neurotrophin signaling pathway
Pyruvate metabolism
Spliceosome
Pyrimidine metabolism
RNA transport
Fanconi anemia pathway
Shigellosis
Other types of O-glycan biosynthesis
Aminoacyl-tRNA biosynthesis
RNA polymerase
Metabolic pathways
Basal transcription factors
TNF signaling pathway
Purine metabolism
Propanoate metabolism
Nucleotide excision repair
Steroid biosynthesis
BT549
Ubiquitin mediated proteolysis
Glycosphingolipid biosynthesis - ganglio series
Peroxisome
FoxO signaling pathway
Wnt signaling pathway
Signaling pathways regulating pluripotency of stem cells
Regulation of autophagy
Glycosylphosphatidylinositol(GPI)-anchor biosynthesis
HIF-1 signaling pathway
Renal cell carcinoma
Fanconi anemia pathway
Neurotrophin signaling pathway
Spliceosome
RNA transport
Chronic myeloid leukemia
Base excision repair
MDA-MB361
Aldosterone synthesis and secretion
Ubiquitin mediated proteolysis
Nucleotide excision repair
-log10(p-value)

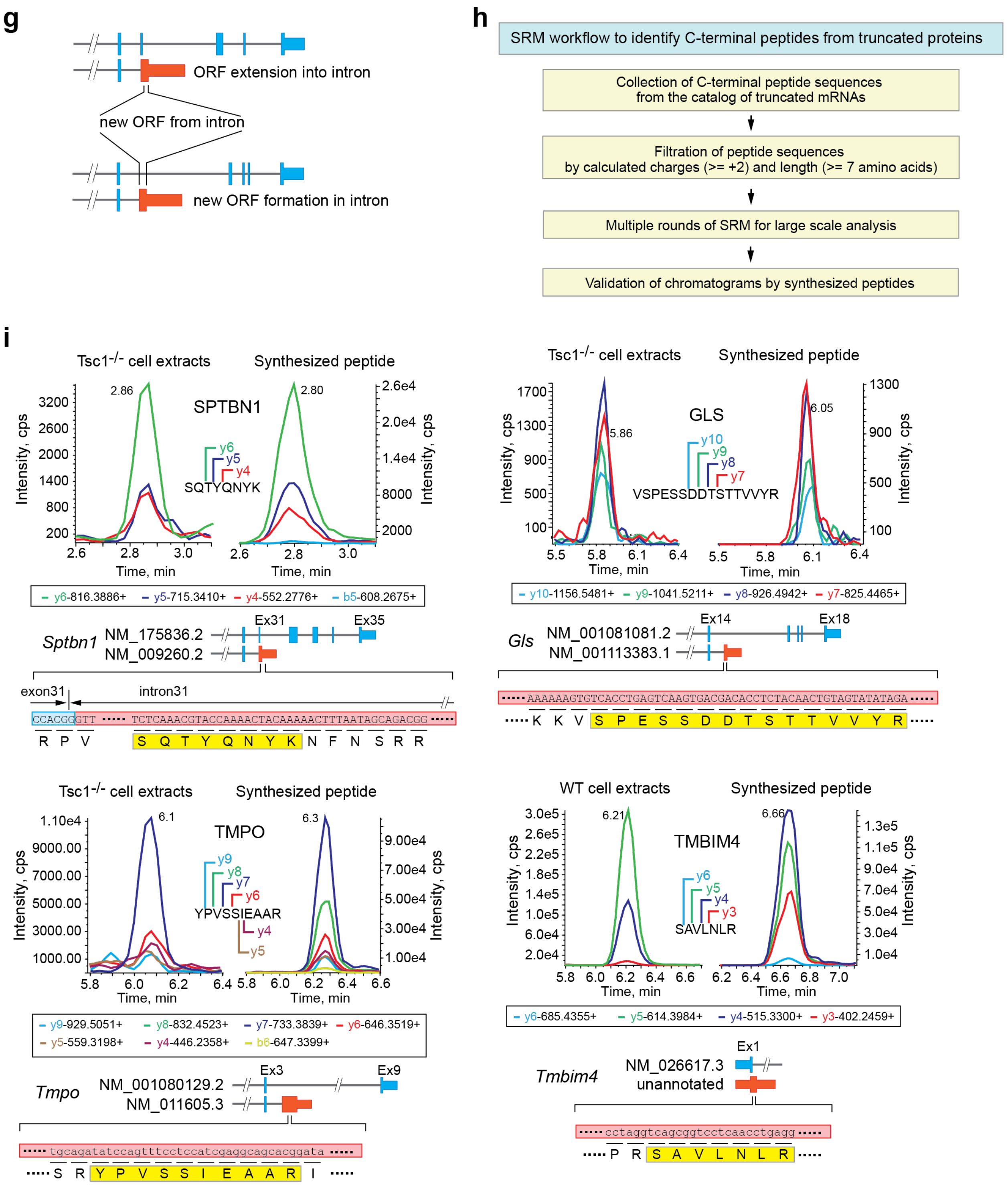

g
ORF extension into intron
new ORF from intron
new ORF formation in intron
h
SRM workflow to identify C-terminal peptides from truncated proteins
Collection of C-terminal peptide sequences from the catalog of truncated mRNAs
Filtration of peptide sequences by calculated charges (>= +2) and length (>= 7 amino acids)
Multiple rounds of SRM for large scale analysis
Validation of chromatograms by synthesized peptides
i
Tsc1-/- cell extracts
Synthesized peptide
SPTBN1
SQTYQNYK
Intensity, cps
Time, min
y6-816.3886+
y5-715.3410+
y4-552.2776+
b5-608.2675+
Sptbn1
NM_175836.2
NM_009260.2
Ex31
Ex35
exon31
intron31
CCACGGGTT ····· TCTCAAACGTACCAAAACTACAAAAACTTTAATAGCAGACGG ·····
R P V S Q T Y Q N Y K N F N S R R
GLS
VSPESSDDTSTTVVYR
y10-1156.5481+
y9-1041.5211+
y8-926.4942+
y7-825.4465+
Gls
NM_001081081.2
NM_001113383.1
Ex14
Ex18
····· AAAAAAGTGTCACCTGAGTCAAGTGACGACACCTCTACAACTGTAGTATATAGA ·····
····· K K V S P E S S D D T S T T V V Y R ·····
TMPO
YPVSSIEAAR
y9-929.5051+
y8-832.4523+
y7-733.3839+
y6-646.3519+
y5-559.3198+
y4-446.2358+
b6-647.3399+
Tmpo
NM_001080129.2
NM_011605.3
Ex3
Ex9
····· tgcagatatccagtttcctccatcgaggcagcacggata ·····
····· S R Y P V S S I E A A R I ·····
WT cell extracts
TMBIM4
SAVLNLR
y6-685.4355+
y5-614.3984+
y4-515.3300+
y3-402.2459+
Tmbim4
NM_026617.3
unannotated
Ex1
····· cctaggtcagcggtcctcaacctgagg ·····
····· P R S A V L N L R ·····

Supplementary Figure 1. a Scatter plots for TR of genes in low and high mTOR in *Tsc1*$^{-/-}$ MEFs. *Tsc1*$^{-/-}$ + mock (high mTOR) vs *Tsc1*$^{-/-}$ + Torin 1 (100 nM, 24 hours; low mTOR) were compared. **b** Additional examples of RNA-Seq read alignments for genes showing enriched intronic APA events in high mTOR *Tsc1*$^{-/-}$ MEFs. The alignments were color-coded as indicated in the figure. The yellow box highlights the regions with the intronic APA event. **c** and **d** Real-time quantitative PCR (RT-qPCR) validation of genes showing dynamic intronic APA events upon the changes of mTOR signaling in cells. RNAi knockdown of mTOR kinase was conducted using *Tsc1*$^{-/-}$ MEFs and the changes of TR for selected genes were tested for intronic APA events. The selection of genes was based on the enriched intronic APA events in *Tsc1*$^{-/-}$ or WT MEFs. The Y-axis scale is shown in the log scale. Four technical repeats were conducted, and students' t-tests were performed for statistical analysis. The data are presented as the mean (SD). **e** Overlap of intronic APA genes across the investigated breast cancer cell lines. Overlapping intronic APA events among three breast cancer cell lines with mock or Torin 1 treatment were analyzed. **f** Enriched KEGG pathways by intronic APA events in three breast cancer cell lines. Enrichment was analyzed by the comparison between mock and Torin 1 treated cells. **g** A schematic presentation of intronic APA and the formation of new ORFs based on intron regions. **h** A SRM workflow for the identification of new peptide sequences from intron regions. **i** Peptide sequences that are produced from intron regions due to intronic APA. Peptides were identified and verified by SRM.

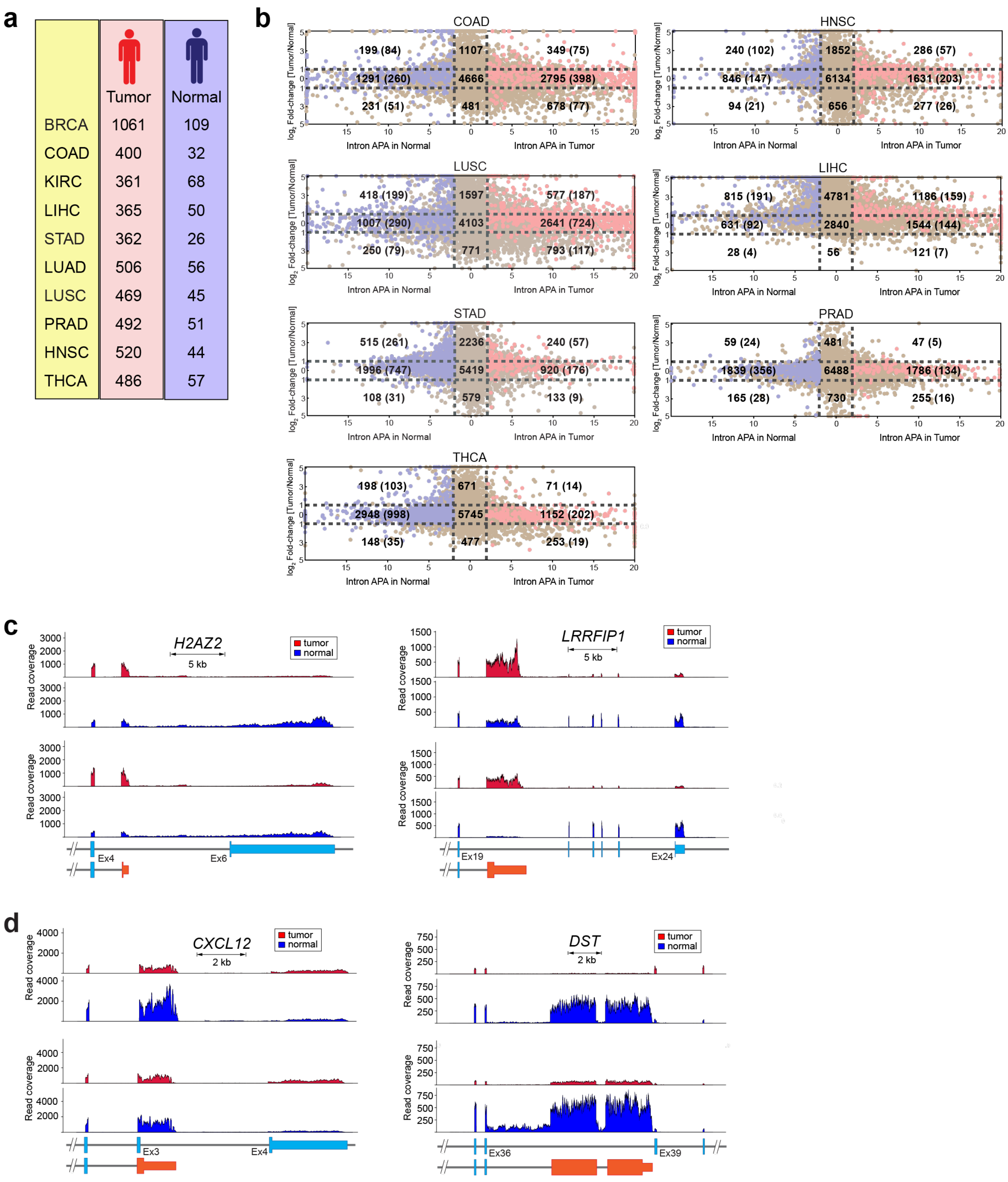
a
Tumor
Normal
BRCA 1061 109
COAD 400 32
KIRC 361 68
LIHC 365 50
STAD 362 26
LUAD 506 56
LUSC 469 45
PRAD 492 51
HNSC 520 44
THCA 486 57
b
COAD
199 (84)
1107
349 (75)
1291 (260)
4666
2795 (398)
231 (51)
481
678 (77)
HNSC
240 (102)
1852
286 (57)
846 (147)
6134
1631 (203)
94 (21)
656
277 (26)
LUSC
418 (199)
1597
577 (187)
1007 (290)
4103
2641 (724)
250 (79)
771
793 (117)
LIHC
815 (191)
4781
1186 (159)
631 (92)
2840
1544 (144)
28 (4)
56
121 (7)
STAD
515 (261)
2236
240 (57)
1996 (747)
5419
920 (176)
108 (31)
579
133 (9)
PRAD
59 (24)
481
47 (5)
1839 (356)
6488
1786 (134)
165 (28)
730
255 (16)
THCA
198 (103)
671
71 (14)
2948 (998)
5745
1152 (202)
148 (35)
477
253 (19)
log2 Fold-change [Tumor/Normal]
Intron APA in Normal
Intron APA in Tumor
c
H2AZ2
5 kb
tumor
normal
Read coverage
Ex4
Ex6
LRRFIP1
5 kb
Ex19
Ex24
d
CXCL12
2 kb
Ex3
Ex4
DST
2 kb
Ex36
Ex39

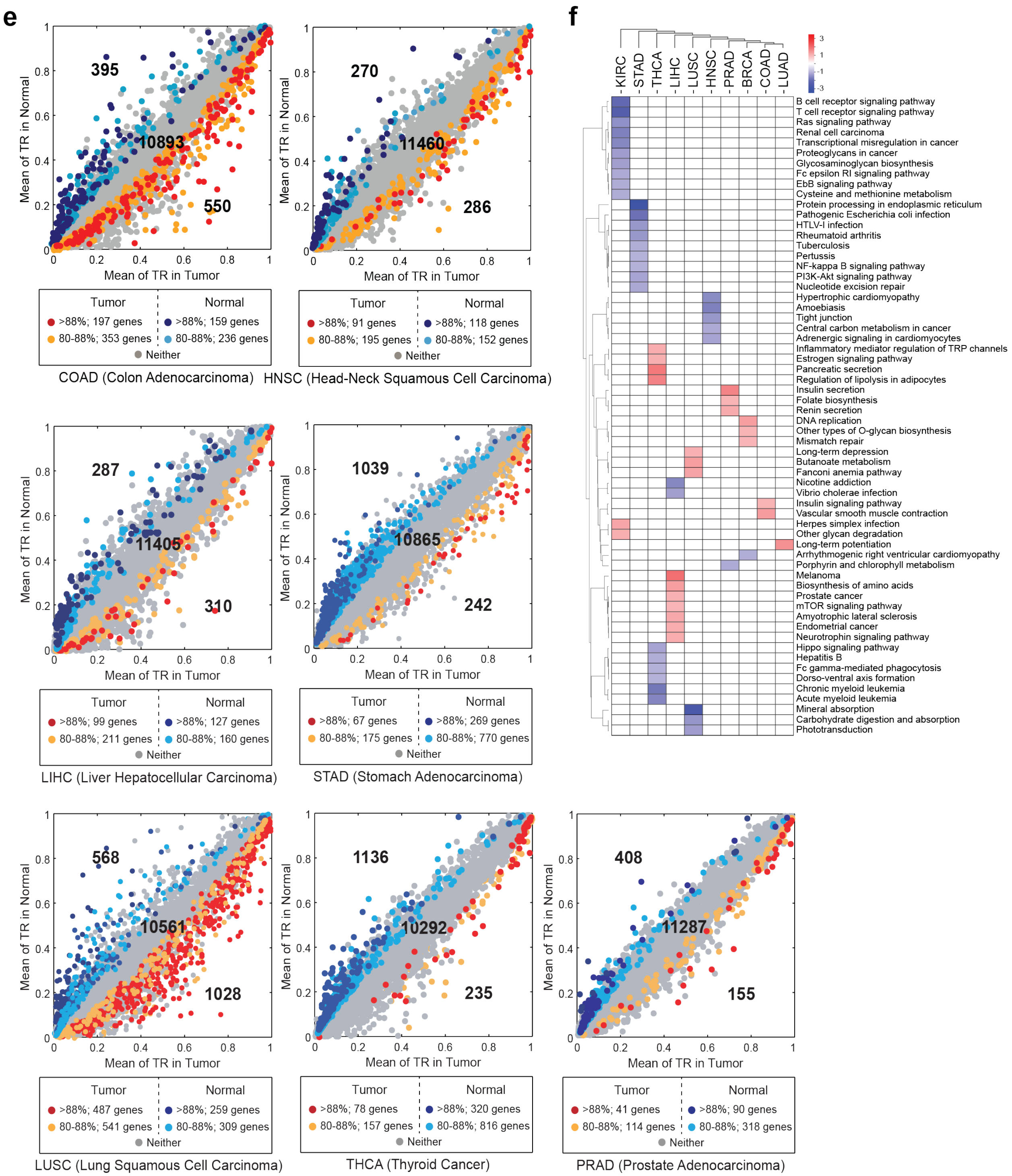
e
Mean of TR in Normal
Mean of TR in Tumor
395
10893
550
Tumor
Normal
>88%; 197 genes
80-88%; 353 genes
>88%; 159 genes
80-88%; 236 genes
Neither
COAD (Colon Adenocarcinoma)
270
11460
286
>88%; 91 genes
80-88%; 195 genes
>88%; 118 genes
80-88%; 152 genes
HNSC (Head-Neck Squamous Cell Carcinoma)
287
11405
310
>88%; 99 genes
80-88%; 211 genes
>88%; 127 genes
80-88%; 160 genes
LIHC (Liver Hepatocellular Carcinoma)
1039
10865
242
>88%; 67 genes
80-88%; 175 genes
>88%; 269 genes
80-88%; 770 genes
STAD (Stomach Adenocarcinoma)
568
10561
1028
>88%; 487 genes
80-88%; 541 genes
>88%; 259 genes
80-88%; 309 genes
LUSC (Lung Squamous Cell Carcinoma)
1136
10292
235
>88%; 78 genes
80-88%; 157 genes
>88%; 320 genes
80-88%; 816 genes
THCA (Thyroid Cancer)
408
11287
155
>88%; 41 genes
80-88%; 114 genes
>88%; 90 genes
80-88%; 318 genes
PRAD (Prostate Adenocarcinoma)
f
KIRC
STAD
THCA
LIHC
LUSC
HNSC
PRAD
BRCA
COAD
LUAD
B cell receptor signaling pathway
T cell receptor signaling pathway
Ras signaling pathway
Renal cell carcinoma
Transcriptional misregulation in cancer
Proteoglycans in cancer
Glycosaminoglycan biosynthesis
Fc epsilon RI signaling pathway
EbB signaling pathway
Cysteine and methionine metabolism
Protein processing in endoplasmic reticulum
Pathogenic Escherichia coli infection
HTLV-I infection
Rheumatoid arthritis
Tuberculosis
Pertussis
NF-kappa B signaling pathway
PI3K-Akt signaling pathway
Nucleotide excision repair
Hypertrophic cardiomyopathy
Amoebiasis
Tight junction
Central carbon metabolism in cancer
Adrenergic signaling in cardiomyocytes
Inflammatory mediator regulation of TRP channels
Estrogen signaling pathway
Pancreatic secretion
Regulation of lipolysis in adipocytes
Insulin secretion
Folate biosynthesis
Renin secretion
DNA replication
Other types of O-glycan biosynthesis
Mismatch repair
Long-term depression
Butanoate metabolism
Fanconi anemia pathway
Nicotine addiction
Vibrio cholerae infection
Insulin signaling pathway
Vascular smooth muscle contraction
Herpes simplex infection
Other glycan degradation
Long-term potentiation
Arrhythmogenic right ventricular cardiomyopathy
Porphyrin and chlorophyll metabolism
Melanoma
Biosynthesis of amino acids
Prostate cancer
mTOR signaling pathway
Amyotrophic lateral sclerosis
Endometrial cancer
Neurotrophin signaling pathway
Hippo signaling pathway
Hepatitis B
Fc gamma-mediated phagocytosis
Dorso-ventral axis formation
Chronic myeloid leukemia
Acute myeloid leukemia
Mineral absorption
Carbohydrate digestion and absorption
Phototransduction

Supplementary Figure 2. a Collection of tumor samples and normal tissues from 10 types of cancer in the TCGA data. **b** Differential expression analyses for genes with annotated intronic APA events in normal tissues and tumor samples. TCGA-COAD (Colon Adenocarcinoma), TCGA-HNSC (Head and Neck Squamous Cell Carcinomas), TCGA-LUSC (Lung Squamous Cell Carcinoma), TCGA-LIHC (Liver Hepatocellular Carcinoma), TCGA-STAD (Stomach adenocarcinoma), TCGA-PRAD (Prostate Adenocarcinoma), and TCGA-THCA (Thyroid carcinoma) data analyses are shown. The x-axis presents the significance of intronic APA events calculated by $-\log_{10}(p\text{-value})$. *p*-values were determined by students' t-test. The y-axis shows the fold changes of gene expression in tumors over normal samples. Red dots indicate the genes showing significant intronic APA events conserved in 80% or more of tumor samples (i.e., 80% or more of tumor samples have higher TRs than the mean TR of normal tissue samples). Blue dots indicate the genes showing significant APA events that 80% or more of tumor samples have lower TRs than the mean TR of normal tissue samples. **c** Exemplary RNA-Seq read alignments from BRCA data for *H2AZ2* and *LRRFIP1* genes. **d** Exemplary RNA-Seq read alignments from BRCA data for *CXCL12* and *DST* genes. **e** Scatter plots for intronic APA events in COAD, HNSC, LUSC, LIHC, STAD, PRAD, and THCA. The TR mean for genes with significant intronic APA events is color-coded. Genes showing intronic APA events in more than 88% of samples are color-coded as blue (normal) or red (tumor). Genes with intronic APA events in 80-88% of samples are shown in cyan (normal) or orange (tumor). **f** A heatmap for the KEGG pathways that are enriched by intronic APA events. The KEGG pathways that are unique to each cancer type are displayed. The color scale represents $-\log_{10}(p\text{-value})$ for pathways enriched in tumor and $\log_{10}(p\text{-value})$ for pathways enriched in normal: red-colored KEGG pathways are enriched in tumor samples and blue-colored KEGG pathways are enriched in normal tissues.

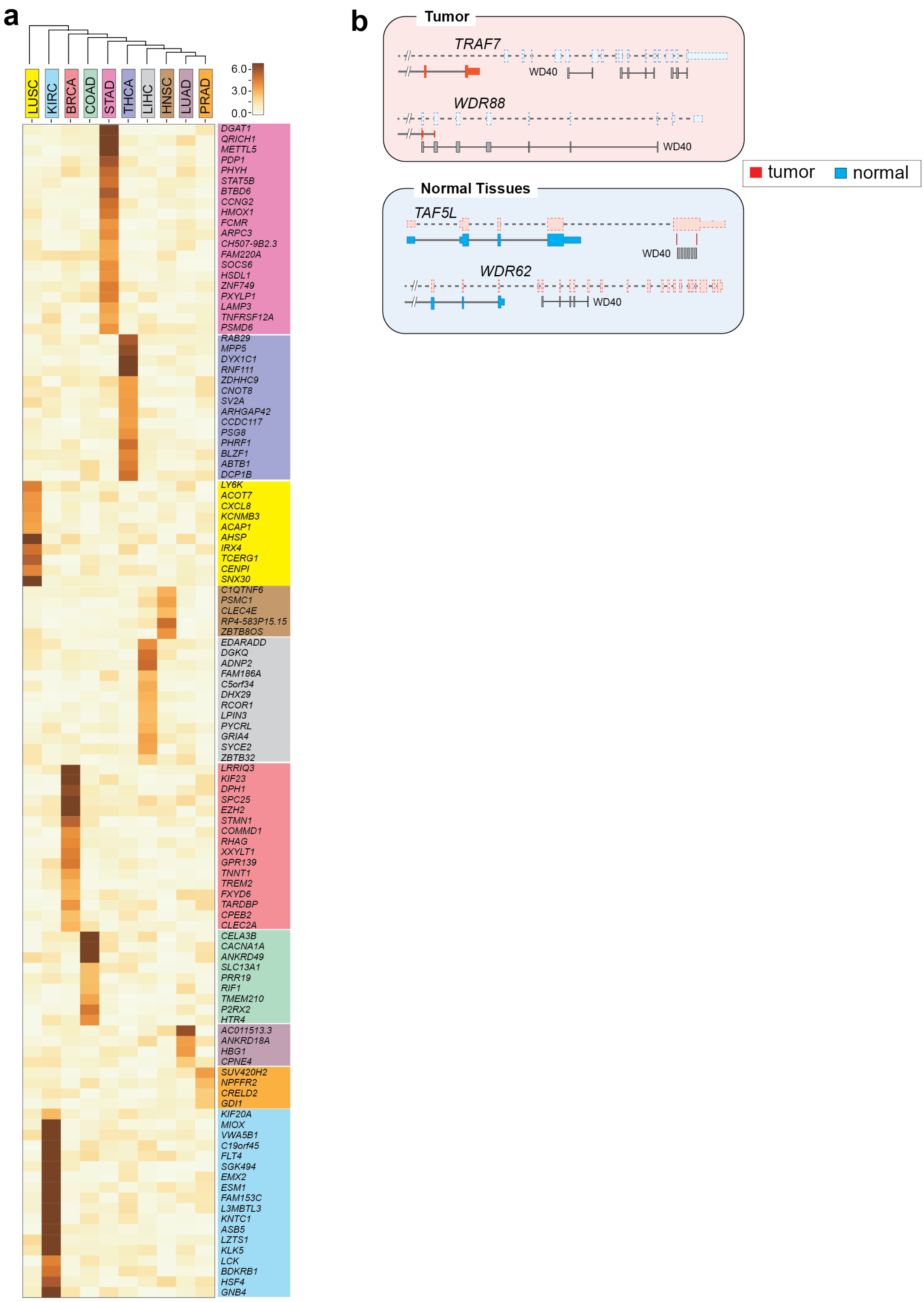

a
LUSC
KIRC
BRCA
COAD
STAD
THCA
LIHC
HNSC
LUAD
PRAD
6.0
3.0
0.0
DGAT1
QRICH1
METTL5
PDP1
PHYH
STAT5B
BTBD6
CCNG2
HMOX1
FCMR
ARPC3
CH507-9B2.3
FAM220A
SOCS6
HSDL1
ZNF749
PXYLP1
LAMP3
TNFRSF12A
PSMD6
RAB29
MPP5
DYX1C1
RNF111
ZDHHC9
CNOT8
SV2A
ARHGAP42
CCDC117
PSG8
PHRF1
BLZF1
ABTB1
DCP1B
LY6K
ACOT7
CXCL8
KCNMB3
ACAP1
AHSP
IRX4
TCERG1
CENPI
SNX30
C1QTNF6
PSMC1
CLEC4E
RP4-583P15.15
ZBTB8OS
EDARADD
DGKQ
ADNP2
FAM186A
C5orf34
DHX29
RCOR1
LPIN3
PYCRL
GRIA4
SYCE2
ZBTB32
LRRIQ3
KIF23
DPH1
SPC25
EZH2
STMN1
COMMD1
RHAG
XXYLT1
GPR139
TNNT1
TREM2
FXYD6
TARDBP
CPEB2
CLEC2A
CELA3B
CACNA1A
ANKRD49
SLC13A1
PRR19
RIF1
TMEM210
P2RX2
HTR4
AC011513.3
ANKRD18A
HBG1
CPNE4
SUV420H2
NPFFR2
CRELD2
GDI1
KIF20A
MIOX
VWA5B1
C19orf45
FLT4
SGK494
EMX2
ESM1
FAM153C
L3MBTL3
KNTC1
ASB5
LZTS1
KLK5
LCK
BDKRB1
HSF4
GNB4
b
Tumor
TRAF7
WD40
WDR88
WD40
tumor
normal
Normal Tissues
TAF5L
WD40
WDR62
WD40

Supplementary Figure 3. a Examples of intronic APA events that are unique to each cancer type. **b** Schematic presentation of Pfam domain swapping in tumors and normal tissues by intronic APA events. The WD-40 domain containing proteins are shown as an example.

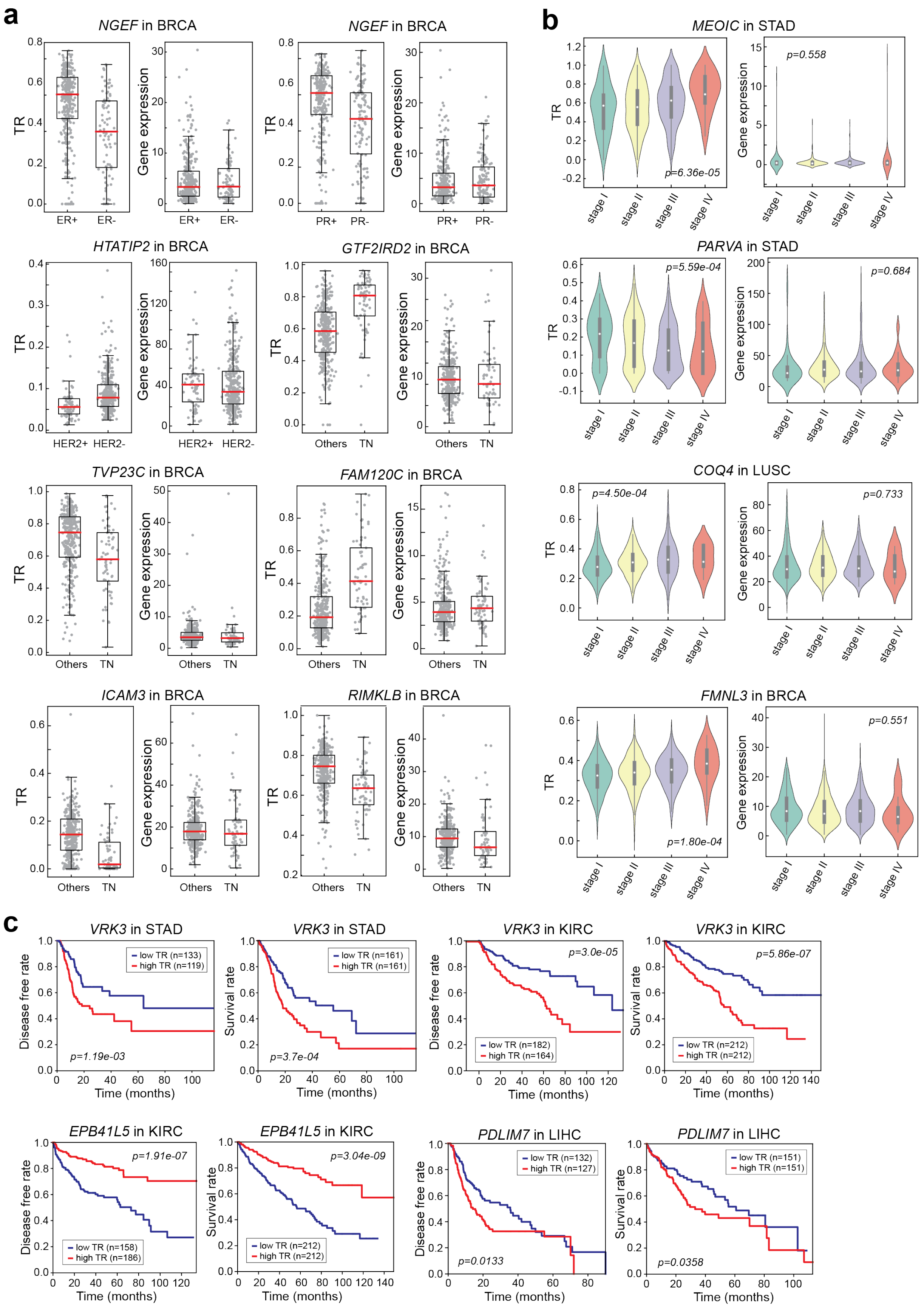

a
NGEF in BRCA
TR
Gene expression
ER+
ER-
PR+
PR-
HTATIP2 in BRCA
HER2+
HER2-
GTF2IRD2 in BRCA
Others
TN
TVP23C in BRCA
FAM120C in BRCA
ICAM3 in BRCA
RIMKLB in BRCA
b
MEOIC in STAD
p=6.36e-05
p=0.558
PARVA in STAD
p=5.59e-04
p=0.684
COQ4 in LUSC
p=4.50e-04
p=0.733
FMNL3 in BRCA
p=1.80e-04
p=0.551
stage I
stage II
stage III
stage IV
c
VRK3 in STAD
low TR (n=133)
high TR (n=119)
p=1.19e-03
Disease free rate
Time (months)
VRK3 in STAD
low TR (n=161)
high TR (n=161)
p=3.7e-04
Survival rate
VRK3 in KIRC
p=3.0e-05
low TR (n=182)
high TR (n=164)
VRK3 in KIRC
p=5.86e-07
low TR (n=212)
high TR (n=212)
EPB41L5 in KIRC
p=1.91e-07
low TR (n=158)
high TR (n=186)
EPB41L5 in KIRC
p=3.04e-09
low TR (n=212)
high TR (n=212)
PDLIM7 in LIHC
low TR (n=132)
high TR (n=127)
p=0.0133
PDLIM7 in LIHC
low TR (n=151)
high TR (n=151)
p=0.0358

Supplementary Figure 4. a The boxplots show four examples that intronic APA events are correlated with hormone receptor phenotypes but not the corresponding gene expression levels in BRCA data. The *p*-values were determined by unpaired t-test. *NGEF* TR *p*=1.25e-10, Gene expression (GE) *p*=0.603; *NGEF* TR *p*=6.60e-8, Gene expression (GE) *p*=0.747; *HTATIP2* TR *p*=1.95e-6, GE *p*=0.964; *GTF2IRD2* TR *p*=6.90e-12, GE *p*=0.540; *TVP23C* TR *p*=3.61e-6, GE *p*=0.721; *FAM120C* TR *p*=2.09e-15, GE *p*=0.536; *ICAM3* TR *p*=9.46e-11, GE *p*=0.802; *RIMKLB* TR *p*=3.36e-10, GE *p*=0.640. **b** Violin plots illustrate multiple exemplary genes demonstrating significant intronic APA events but not significant differential gene expression in cancer stages. **c** Kaplan-Meier (KM) plots illustrate the correlation between the TR of selected genes (*VRK3, EPB41L5* and *PDLIM7*) and the disease-free rate or survival rate of cancer patients in STAD, LIHC and KIRC.

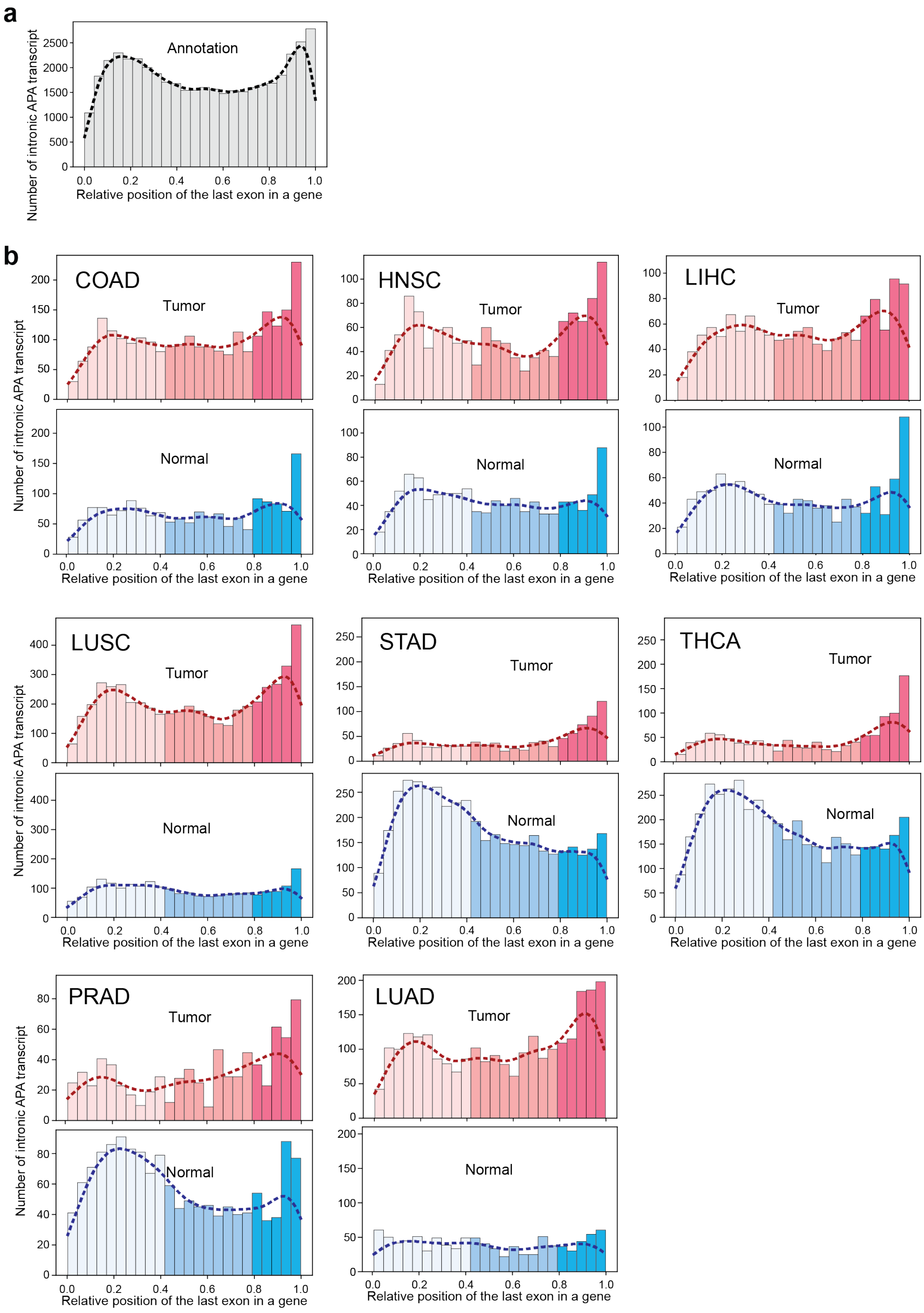

a
Annotation
Number of intronic APA transcript
Relative position of the last exon in a gene
b
COAD
HNSC
LIHC
LUSC
STAD
THCA
PRAD
LUAD
Tumor
Normal
Number of intronic APA transcript
Relative position of the last exon in a gene

Supplementary Figure 5. a The distribution of relative positions of the 3'-last exon of intronic APA transcripts. The graph was generated using all annotated intronic APA transcripts and their corresponding full-length transcripts registered in the current genome annotation. **b** Histogram displays for the distribution of intronic APA position in pan-cancer data.
